# Surface melting–driven hydrogen absorption for high-pressure polyhydride synthesis

**DOI:** 10.1073/pnas.2413480122

**Published:** 2025-05-29

**Authors:** Ryuhei Sato, Lewis J. Conway, Di Zhang, Chris J. Pickard, Kazuto Akagi, Kartik Sau, Hao Li, Shin-ichi Orimo

**Affiliations:** ^a^Advanced Institute for Materials Research, Tohoku University, Sendai 980-8577, Japan; ^b^Department of Materials Engineering, The University of Tokyo, Tokyo 113-8656, Japan; ^c^Department of Materials Science and Metallurgy, University of Cambridge, Cambridge CB3 0FS, United Kingdom; ^d^Mathematics for Advanced Materials Open Innovation Laboratory, National Institute of Advanced Industrial Science and Technology, c/o Advanced Institute for Materials Research, Tohoku University, Sendai 980-8577, Japan; ^e^Institute for Materials Research, Tohoku University, Sendai 980-8577, Japan

**Keywords:** polyhydride, high pressure, surface melting, molecular dynamics, machine learning potential

## Abstract

The synthesis of new polyhydrides with high superconducting *T*_c_ is challenging owing to the high pressures and temperatures required. In this study, we used machine-learning potential molecular dynamics simulations to investigate the initial stage of polyhydride formation in calcium hydrides. Upon contact with high-pressure H_2_, the surface of CaH_2_ melts, leading to CaH_4_ formation. This surface melting proceeds via CaH_4_ liquid phase as an intermediate state. High pressure reduces not only the hydrogenation (CaH_2_(s) + H_2_(l) ↔ CaH_4_(s)) enthalpy but also the enthalpy for liquid polyhydride formation (CaH_2_(s) + H_2_(l) ↔ CaH_4_(l)). Consequently, this surface melting process becomes more favorable than the fusion of the polyhydride bulk. Thus, high pressure not only shifts the equilibrium toward the polyhydride product but also lowers the activation energy, thereby promoting the hydrogenation reaction. From these thermodynamic insights, we propose structure-search criteria for polyhydride synthesis that are both computationally effective and experimentally relevant. These criteria are based on bulk properties, such as polyhydride (product) melting temperature and pressure-dependent hydrogenation enthalpy, readily determined through supplementary calculations during structure prediction workflows.

## Significance

Room-temperature superconductors could revolutionize modern technology by enabling lossless power transmission, powerful magnets, advanced medical imaging (MRI), and quantum computing. Polyhydrides—hydrogen-rich compounds synthesized under extreme pressures—are among the most promising candidates. However, their synthesis mechanisms remain poorly understood, creating a bottleneck in materials discovery. Here, we apply machine learning potentials to perform large-scale molecular dynamics simulations of solid/hydrogen interfacial reactions under high pressure, involving thousands of atoms. Our results reveal how pressure kinetically promotes the reaction and suggest principles that extend beyond hydrides to pressure-driven chemical synthesis more broadly. This work demonstrates the ability of machine learning potentials to uncover complex interfacial reaction mechanisms, opening an avenue toward predictive reaction modeling in materials science.

Since the discovery of superconductivity in mercury by Onnes in 1911 (1), Room-temperature superconductivity is one of the “holy grails” of physics. Its realization promises not only fundamental insights but also major societal impact. Eliminating energy loss from electrical resistance could transform power systems. Superconductors are also essential in quantum computing [e.g., IBM Quantum (2), Google Willow (3)], MRI (4), and next-generation electronic and magnetic devices. However, current superconductors operate only below room temperature. Developing materials operating at or above room temperature remains a key challenge for practical applications, such as everyday electric power cables or consumer-accessible quantum computer.

Among superconducting materials, polyhydrides (5–17) have shown the highest *T*_c_ values and are considered strong candidates for room-temperature superconductivity. Binary hydrides with high hydrogen-to-metal (H/M) ratios, such as LaH_10_ (7–10) and CaH_6_ (11–13), have demonstrated *T*_c_ values of 250 to 260 K at 180 GPa and 210 K at 160 GPa, respectively. Ternary and quaternary polyhydrides may further enhance *T*_c_ (18–21). These materials are typically predicted via DFT-based structure search methods [e.g., random (20, 21) or metaheuristic approaches (7, 11, 13–16, 18)] and subsequently synthesized under high pressures using diamond anvil cells (DACs).

Despite these successes, polyhydride synthesis remains challenging. For instance, it took nearly a decade to experimentally realize CaH_6_ (12, 13) after its theoretical prediction (11). A major hurdle is the extremely high pressures required for stabilization. In addition, in most cases, laser heating is also necessary to overcome kinetic barriers, except for certain materials like LaH_10_ (10) and CeH_9_ (16). These observations suggest that polyhydride formation involves slow reaction kinetics or high activation energies. Recently, electrochemical synthesis under pressure has been proposed to reduce stabilization pressures (22). However, most efforts still rely on thermal synthesis. Experimental characterization in DACs is limited to techniques like XRD and Raman spectroscopy, and direct theoretical modeling by DFT calculations is hindered by the large system sizes involved. As a result, the reaction dynamics of hydrogenation remain poorly understood, and guidelines for efficient polyhydride synthesis are lacking.

Thus, theoretical materials science now faces a grand challenge: not only to predict structures and properties but also to understand and predict the synthesis routes of materials. This challenge is not limited to polyhydrides but applies broadly across materials. For example, a study comparing experimentally reported compounds with DFT-based stability assessments found that a substantial fraction are metastable—slightly higher in energy than the most stable phases (23). Such metastable materials are often synthesized to achieve specific functionalities. As a result, identifying feasible synthetic pathways for theoretically predicted compounds remains a major bottleneck in materials discovery. The structural search method combined with machine learning potentials (MLPs) enabled to propose the synthetic route of predicted materials from the viewpoint of the thermodynamics, as in the case of Mg_2_IrH_6_ (21). However, the workflow taking into account the kinetics is essential for proposing more accurate synthetic routes.

This grand challenge is expected to be achieved through molecular dynamics (MD) simulations and the latest machine learning methods. MD simulations are one of the most effective computational methods for the direct observation of dynamic phenomena. For example, MD simulations with interatomic potentials have contributed to the elucidation of phenomena such as superionic conduction mechanisms (24, 25), carbon nanotube formation (26), and protein folding (27). The recent developments of MLPs (28–34) have accelerated and facilitated the potential construction of new materials. Machine learning interatomic potentials, first introduced over a decade ago (28), have recently become broadly accessible and highly accurate, driven by advances in software implementations (30, 32–34). These developments have enabled MD simulations to be applied to a wider variety of materials (20, 21, 35–40). In fact, MLP-MD simulations have accurately reproduced the phase transition behavior of high-pressure H_2_, which is strongly affected by the simulation cell size (35). In addition, MLPs are frequently used to explore the bulk properties of hydrides (36, 37) and the structure of ternary systems (20, 21). However, the application of MLPs has so far been mainly limited to homogeneous systems. Although some studies have reported applications to inert heterogeneous interfaces such as solid/water boundaries (39, 40), there appear to be few examples that describe chemically reactive interfaces in which the solid phase itself undergoes transformation.

In this study, to provide a physical insight into the polyhydride synthesis, we addressed the challenge of MLP construction for unknown heterogeneous chemical reactions and directly observed CaH_2_ hydrogenation by MLP-MD simulations. CaH_2_ is an ionic crystal composed of Ca^2+^ and H^−^ with an hcp Ca lattice. On the other hand, CaH_4_ (41–43) forms an fcc Ca lattice, which contains H^−^ ions and H_2_^δ−^ dimers. Therefore, the CaH_2_ hydrogenation reaction, CaH_2_ + H_2_ ↔ CaH_4_, is employed as an example of polyhydride synthesis that requires 1) metal lattice reconstruction and 2) hydrogenation starting from stoichiometric ionic crystals whose anion sites are fully occupied by H^−^. Note that CaH_4_ is stable above 20 GPa based on the formation enthalpy from DFT calculations (*SI Appendix*, Fig. S1). However, previous experimental studies (41–43) synthesized CaH_4_ above 50 GPa. Moreover, after synthesis, CaH_4_ becomes stable at around 10 GPa (43). These results suggest the presence of a pressure gap between the synthesis conditions and the thermodynamic equilibrium state, which may be indicative of a significant kinetic barrier for the hydrogenation process. This gap highlights the critical role of pressure not only in determining phase stability but also in facilitating the reaction pathway, especially in solid-state systems where atomic rearrangements are required.

## Results

### CaH_2_ Surface Melting and Subsequent Hydrogenation to Form CaH_4_.

**Fig. 1.**
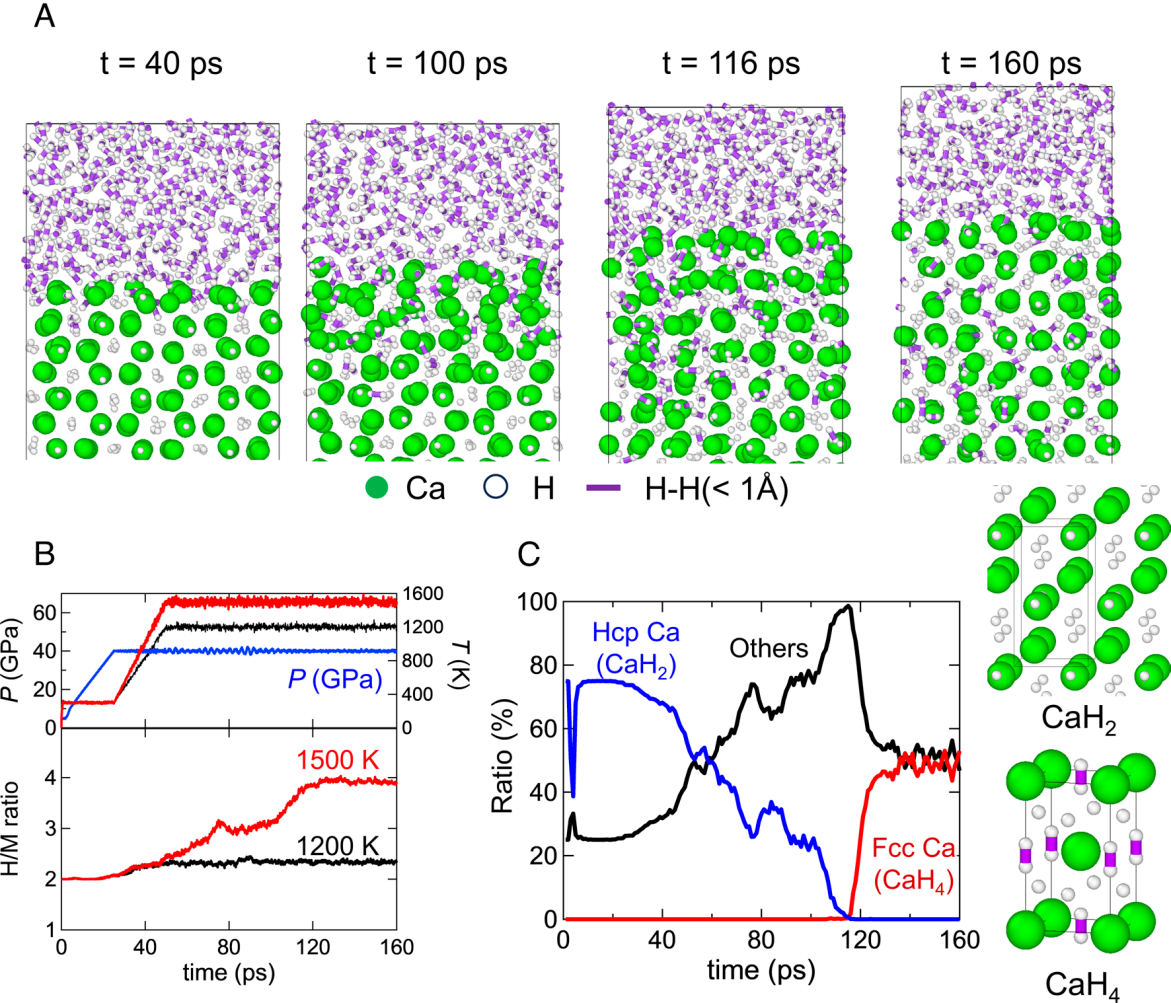
CaH_2_ hydrogenation via surface melting. (*A*) Snapshots of atomic configurations during a MLP-MD simulation of the CaH_2_(100)/H_2_ interface at 1,500 K under 40 GPa. (*B*) Time series of temperature (*T*), pressure (*P*), and H/M ratio during MLP-MD simulations of the CaH_2_(100)/H_2_ interface at (black) 1,200 K and (red) 1,500 K at 40 GPa. Here, if the distance between Ca and H is shorter than 3 Å, the Ca atom is considered coordinated to the H atom. We counted the number of H atoms whose Ca coordination number is greater than three. The H/M ratio in this figure represents the number ratio of these specific H atoms to Ca atoms in the simulation cell. The cutoff distance 3 Å has been chosen on the basis of the first peak of the pair distribution function of H atoms around Ca atoms in *SI Appendix*, Fig. S9. (*C*) Time series of Ca structure ratio (hcp, fcc, others) obtained by a-CNA during MLP-MD simulation of the CaH_2_(100)/H_2_ interface at 1,500 K under 40 GPa. Here, only Ca atoms were used in a-CNA. Note that surface and subsurface Ca atoms are classified as “Others” owing to the lack of neighboring Ca atoms near the surface.

CaH_2_ hydrogenation occurs via surface melting at high temperatures. Fig. 1*A* shows snapshots of a MLP-MD simulation of the CaH_2_(100) surface in contact with high-density H_2_ at *P* = 40 GPa and *T* = 1,500 K. Fig. 1*B* shows the time series of temperature, pressure, and H/M ratio during this MLP-MD simulation. This NPT-MD simulation started at 300 K under 10 GPa so that CaH_2_ does not react with H_2_ molecules during the initial relaxation. Then, the system was pressurized to 40 GPa and heated to 1,500 K for about 50 ps. At *P* = 40 GPa and 1,500 K, the surface structure was disordered (ex. t = 100 ps and 116 ps) to absorb H atoms into the bulk. As shown by the adaptive common neighbor analysis, a-CNA (44), in Fig. 1*C*, Ca in this disordered structure formed neither an hcp Ca nor an fcc Ca lattices. That is, CaH_x_ during hydrogenation formed an amorphous or liquid-like structure rather than solid CaH_2_ or CaH_4_. During this structural disordering, the H/M ratio continued to increase, as shown in Fig. 1*B*. H absorption is also confirmed by the purple lines in the bulk in the MD simulation snapshots. This is because CaH_2_ does not contain any H_2_-molecule-like dimers (H_2_^δ−^), whereas CaH_4_ contains them. Significant Ca rearrangement during hydrogen absorption was also observed in ab initio MD simulation (*SI Appendix*, Fig. S2). This result confirms that the hydrogenation reaction involving structural disordering in our MLP-MD simulation does not reflect the artificial phenomenon induced by the training process or dataset for MLP construction. After this disordered structure absorbed H atoms, it started to form an ordered structure including H_2_^δ–^ (t = 116 and 160 ps.). This is confirmed by the a-CNA results in Fig. 1*C*, showing that the fcc Ca ratio (≈CaH_4_) increased after 120 ps. The fcc Ca ratio was underestimated due to the large thermal oscillation at 1,500 K. In fact, when this calculation cell was quenched to 300 K, the fcc Ca ratio increased to 86 %. The progress of the CaH_2_ hydrogenation reaction is also confirmed by the H/M ratio during the MLP-MD simulation in Fig. 1*B*. The H/M ratio reached 4 at around 120 ps, indicating that almost all CaH_2_ reacts with H_2_ to form CaH_4_. The black line in Fig. 1*B* shows the results of MLP-MD simulation at *T* = 1,200 K. During the MD simulation, the H/M ratio slightly increased due to the H_2_ adsorption on the surface and H_2_ absorption in the subsurface layer. However, the H/M ratio was still close to 2, showing that the surface melting and subsequent hydrogenation did not occur at *T* = 1,200 K.

**Fig. 2.**
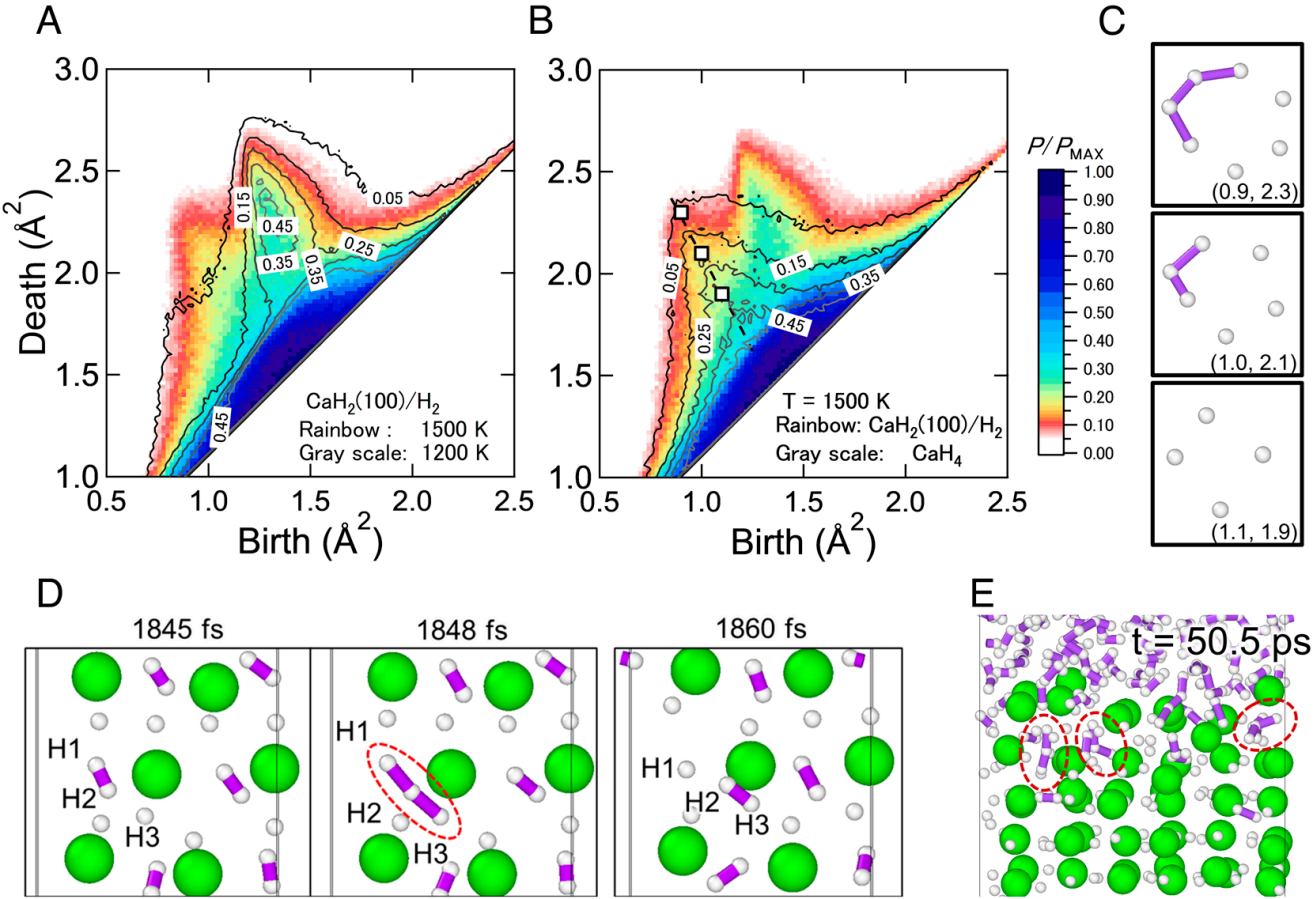
Local structural analysis during hydrogenation by topological data analysis. (*A* and *B*) Time-averaged PDs calculated using H atoms near the CaH_x_ bulk during MLP-MD simulations. PDs were created with only H atoms whose distance to the nearest Ca is shorter than 3 Å. Rainbow color density plots in *A* and *B* show PDs of MLP-MD simulation for the CaH_2_(100)/H_2_ interface at 1,500 K under 40 GPa between 25 and 160 ps. On the other hand, grayscale contours represent MLP-MD simulations for (*A*) the CaH_2_(100)/H_2_ interface at 1,200 K under 40 GPa between 25 and 160 ps and (*B*) the CaH_4_ bulk at 1,500 K under 40 GPa. (*C*) Averaged ring structures for white (birth, death) meshes in *B* obtained by inverse analysis of PD. Purple lines represent H–H bonds with the distance shorter than 1.5 Å. A longer H–H distance was employed to account for analytical errors in calculating these rings. (*D* and *E*) Snapshots of atomic configurations (*D*) during AIMD simulation for the CaH_4_ bulk at 1,000 K under 25 GPa and (*E*) the CaH_2_(100)/H_2_ interface during MLP-MD simulation at 1,500 K under 40 GPa. Here, H–H distance is set to be 1.1 Å.

The local structure around H atoms during surface melting is similar to that around H atoms in CaH_4_, providing an increased number of absorption sites compared with local environments for H atoms in CaH_2_. Fig. 2*A* shows a time-averaged persistence diagram (PD) (45–47) of the MLP-MD simulation of the CaH_2_(100)/H_2_ interface at 1,500 K under 40 GPa with surface melting (rainbow color density plot) compared with that at 1,200 K under 40 GPa without surface melting (grayscale contour plot). Here, H atoms with a minimum Ca–H distance of 3 Å or shorter were included in this persistent homology analysis. Namely, these PDs focus only on the rings (one-dimensional hole in homology) formed by H atoms in CaH_x_ and H atoms adsorbed on the surface. In Fig. 2*A*, new ring structures appear around (birth, death) = (b, d) = (1.0, 2.1) only when CaH_2_ hydrogenation occurs. This shows that these rings are important for the CaH_2_ hydrogenation reaction. Fig. 2*B* shows a PD of the MLP-MD simulation for the CaH_2_(100)/H_2_ interface at 1,500 K under 40 GPa (rainbow color density plot) compared with that for bulk CaH_4_ at 1,500 K under 40 GPa (grayscale contour plot). As shown in Fig. 2*B*, the rings in bulk CaH_4_ are in good agreement with these new rings obtained during CaH_2_ hydrogenation. Therefore, we conclude that during the CaH_2_ hydrogenation, liquid CaH_4_, rather than liquid CaH_2_, formed as an intermediate state. This is further confirmed by the time series of PDs during MLP-MD simulation, since these rings appear before 120 ps, the time before Ca atoms form an fcc lattice (*SI Appendix*, Fig. S3), showing that the local structure for H atoms in amorphous CaH_x_ is similar to those in bulk CaH_4_. Thus, the H content in the bulk increased during hydrogenation owing to the formation of H absorption sites in CaH_4_. Note that this MLP distinguishes between liquid CaH_4_ and the molten state, the mixture of Ca, H, and H_2_. During a 0.5-ns MLP-MD simulation at 40 GPa and 2,800 K, conducted for the CaH_4_/H_2_ interface obtained after the hydrogenation reaction, the Ca and H atoms were completely mixed (*SI Appendix*, Fig. S4). In contrast, at 2,400 K, the interface between liquid CaH_4_ and H_2_ remained intact during the same simulation time (*SI Appendix*, Fig. S4). These results indicate that the hydrogenation reaction proceeds via liquid CaH_4_ as an intermediate phase, rather than through a molten state.

Hydrogenation reactions via liquid or amorphous phases are consistent with previous studies including the experimental observations. Siska et al. reported that yttrium hydride melted during high-pressure synthesis of YH_x_ (x = 4.00 to 5.75) with Weaire–Phelan-like structure from fcc YH_3_ using X-ray free-electron lasers (17). This experimental study agrees well with our MLP-MD simulations, both demonstrating the critical role of melting in facilitating polyhydride formation. Additionally, hydrogen-induced amorphization has been observed in AB_2_-type hydrogen storage materials (48). In *Pnma*-type CaH_2_, doping with La leads to the formation of fluorite-type Ca_0.84_La_0.16_H_2.12_ near the melting point of CaH_2_, suggesting disordering similar to surface melting (49). Furthermore, the surface melting or amorphization has also been observed in aluminum oxidation reactions during MD simulations with a ReaxFF potential (50). This phenomenon was confirmed by in situ TEM observations due to the slow kinetics of the reaction. Similarly, in MgH_x_ systems (x < 2), MLP-MD simulations have confirmed the formation of liquid-like structures during phase separation (37). These findings collectively support the validity of our simulation results, demonstrating that surface melting is a common phenomenon across various material systems.

The chain-like structure of H atoms is important for H^−^ diffusion and absorption during surface melting. Fig. 2*C* shows the averaged ring structures in each (b, d) mesh obtained by the inverse analysis of PDs (51). The ring structure of H atoms obtained by surface melting contains chain-like structures such as H trimers (H_3_^−^) and H tetramers. These structures reflect H^−^ migration paths via Frenkel defects. Fig. 2*D* shows snapshots of H^−^ migration in the CaH_4_ bulk during AIMD simulation at 1,000 K under 25 GPa. H^−^ migrates via H_3_^−^, consistent with the H-chain structure obtained from the PDs derived from MLP-MD. The H diffusion coefficient in CaH_4_ is much larger than in CaH_2_ at 1,500 K, as shown in the Arrhenius plot for H^−^ diffusivity (*SI Appendix*, Fig. S5). This implies that H^−^ migration via H_3_^−^ (Fig. 2*D*) occurs frequently during MD simulations. Such a chain structure of H atoms is also important for interfacial reactions. Fig. 2*E* shows a snapshot near the CaH_2_(100)/H_2_ interface during the MLP-MD simulation at 1,500 K under 40 GPa. Similar to H^−^ migration in CaH_4_, H absorption near the interface is also mediated by H_3_^−^.

Such H^−^ migration via H_3_^−^,

[1] $$H_{H}^{-}+H_{2}^{\delta -}\overset{H_{3}^{-}}{↔}H_{2}^{\delta -}+H_{H}^{-},$$

is regarded as that with a Frenkel defect since the middle H atom in H_3_^−^ is in the interstitial site. A similar H migration mechanism is also reported in LaH_10_ (52). Here, H atoms form H dimers (or two H atoms in interstitial sites) to form vacancy sites. On the other hand, the H^−^ migration mechanisms of bulk CaH_2_ (53) and BaH_2_ (54) are different, because they are based on Schottky defects and use vacancy sites for H^−^ migration in accordance with the following reaction:

[2] $$H_{H}^{\times }↔1/2H_{2}+V_{H}^{\cdot }.$$

Since polyhydride synthesis has been conducted under high $P_{H_{2}}$, the $V_{H}^{\cdot }$ concentration is expected to be low. Thus, the H^−^ diffusion property under ambient pressure is not necessarily applicable as a criterion for polyhydride synthesis under high pressure. Indeed, at lower pressures and temperatures such as 15 GPa and 1,200 K, where no surface melting occurs, H_2_ dissociation on the CaH_2_ surface and subsequent H^–^ migration into the CaH_2_ bulk were observed but did not contribute to H absorption to form CaH_4_, as shown in *SI Appendix*, Fig. S6. It is also noteworthy that the charge separation during the reaction in Eq. **1** does not present a significant barrier. The activation energy for Eq. **1**, calculated from the Arrhenius plot (*SI Appendix*, Fig. S5), was 0.46 eV, which is comparable to that of H^–^ superionic conductors under ambient pressure (55). This result suggests that H diffusion after surface melting is not the rate-limiting step in the hydrogenation process.

### Thermodynamics of Surface Melting and Synthesis Criteria.

**Fig. 3.**
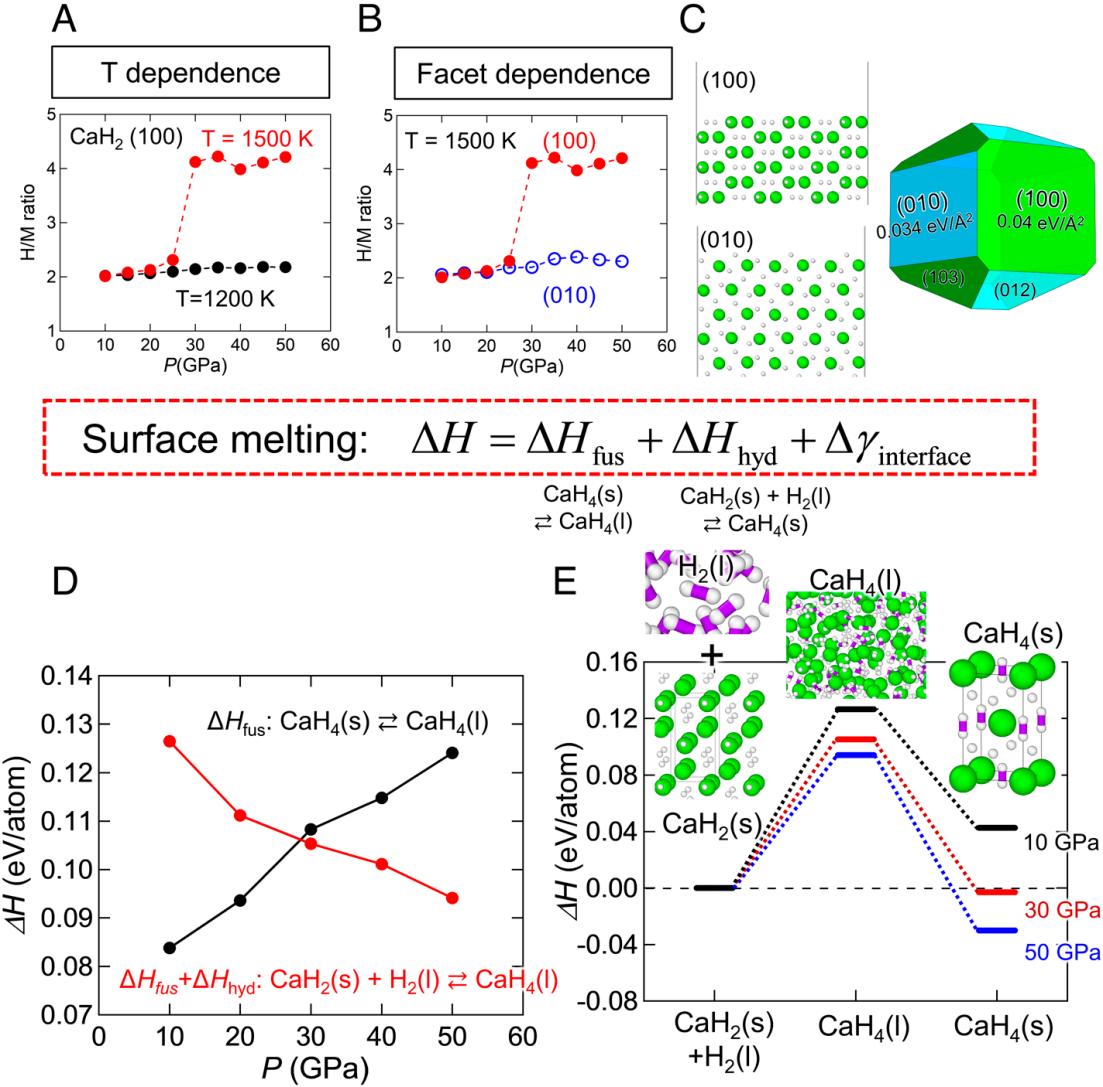
Thermodynamics of CaH_2_ surface melting. H/M ratio of (*A*) CaH_2_(100)/H_2_ interface at 1,200 K (black) and 1,500 K (red) and (*B*) CaH_2_(100)/H_2_ (red) and CaH_2_(010)/H_2_ (blue) interfaces at 1,500 K as a function of *P* (GPa) after 1-ns MLP-MD simulations. (*C*) Wulff shape (i.e., the nanoparticle structure minimizing the surface energy) for CaH_2_ under vacuum. (*D*) Reaction enthalpy per atom as a function of pressure. (*E*) Enthalpy change during CaH_2_ hydrogenation relative to that for solid CaH_2_(s) + H_2_(l) under 10, 30, and 50 GPa. Note that enthalpies at 0 K in *D* and *E* are obtained by the extrapolation of time-averaged enthalpies during 100-ps MLP-MD simulations for the corresponding materials at various temperatures (*SI Appendix*, Figs. S7 and S8).

Fig. 3 *A* and *B* shows the pressure dependence of the H/M ratio after 1-ns MLP-MD simulations. Here, (100) and (010) surfaces were analyzed on the basis of the Wulff shape (i.e., the nanoparticle structure minimizing the surface energy) calculated from the surface energy in vacuum (Fig. 3*C*). From Fig. 3*A*, it is confirmed that the CaH_2_ hydrogenation reaction occurs at 1,500 K above 30 GPa in this MLP. Conversely, hydrogenation does not occur at 1,200 K at any pressure. This indicates that surface melting is a thermally activated process, whose reaction temperature is between 1,200 and 1,500 K. The synthesis conditions in our MLP-MD simulations are comparable with experimental results as summarized in *SI Appendix*, Table S1. In previous studies, CaH_4_ was synthesized at temperatures above 1,600 K and pressures above 50 GPa (41–43). The formation enthalpy calculated using DFT (VASP) was –0.47 eV/unit at 60 GPa, while our MLP model predicted the same value at 30 GPa. This difference suggests that the MLP model predicts the synthesis pressure at a slightly lower range. However, after considering the DFT pressure scale, the simulation conditions show good agreement with experimental observations, demonstrating the model's reliability in reproducing experimental trends.

Fig. 3*B* shows the H/M ratios after 1-ns MLP-MD simulations for CaH_2_(100)/H_2_ and CaH_2_(010)/H_2_ interfaces under various pressures at 1,500 K. Hydrogenation does not occur near the CaH_2_(010)/H_2_ interface at any pressure. This result is associated with the surface energies of (100) and (010). The surface energy of (010) in vacuum is determined to be 0.034 eV/Å^2^ by DFT calculation, which is lower than 0.04 eV/Å^2^ of (100). Thus, although this is a qualitative discussion based on the surface energy under vacuum conditions, this hydrogenation behavior suggests that the surface melting is affected by the interfacial energy between CaH_2_ and H_2_.

Hydrogenation via surface melting can be explained by the following thermodynamic argument. The conventional equation for surface melting for CaH_4_ without hydrogenation is as follows,

[3] $$\begin{array}{c} \Delta G=\Delta G_{\mathrm{fus}}+\Delta \gamma_{\mathrm{interface}};\;\Delta \gamma_{\mathrm{interface}}=\gamma_{{\mathrm{CaH}}_{4}\left(s\right)-{\mathrm{CaH}}_{4}(l)}\\ +\gamma_{{\mathrm{CaH}}_{4}\left(l\right)-H_{2}(l)}-\gamma_{{\mathrm{CaH}}_{4}\left(s\right)-H_{2}(l)}. \end{array}$$

On the other hand, when CaH_2_ hydrogenation reactions are involved,

[4] $$\begin{array}{c} \Delta G=\Delta G_{\mathrm{fus}}+\Delta G_{\mathrm{hyd}}+\Delta \gamma_{\mathrm{interface}};\;\Delta \gamma_{\mathrm{interface}}\\ \;=\gamma_{{\mathrm{CaH}}_{2}\left(s\right)-{\mathrm{CaH}}_{4}(l)}\\ \;+\gamma_{{\mathrm{CaH}}_{4}\left(l\right)-H_{2}(l)}-\gamma_{{\mathrm{CaH}}_{2}\left(s\right)-H_{2}(l)}. \end{array}$$

In this equation, Δ*G*_fus_ denotes the Gibbs free energy of CaH_4_ fusion (CaH_4_(s) ↔ CaH_4_(l)), whereas Δ*G*_hyd_ represents that of the CaH_2_ hydrogenation reaction, CaH_2_(s) + H_2_(l) ↔ CaH_4_(s). The symbols (s) and (l) indicate the solid and liquid phases, respectively. γ_A-B_ represents the energy of the interface between A and B. Since Δ*G*_hyd_ becomes negative under high pressure, where the hydrogenation reaction occurs, the surface melting is promoted, as described by Eq. **4**. As a first approximation, here, we focus only on the enthalpies of the homogeneous system of CaH_2_, CaH_4_, and H_2_, neglecting the entropy and interfacial energy contributions. Fig. 3*D* shows the pressure dependence of the enthalpies for CaH_4_ fusion (Δ*H*_fus_) and liquid CaH_4_ formation by CaH_2_ hydrogenation (Δ*H*_fus_+Δ*H*_hyd_). The time-averaged enthalpies derived from the 100-ps MLP-MD simulations at various temperatures were fitted and extrapolated to determine the enthalpy at 0 K (*SI Appendix*, Figs. S7 and S8), which was employed in these reaction enthalpy calculations. As shown in Fig. 3*D*, Δ*H*_fus_+Δ*H*_hyd_ is lower than Δ*H*_fus_ at pressures above 30 GPa. Consequently, surface melting via hydrogenation could occur at temperatures below the melting point of CaH_4_ at pressures over 30 GPa. Therefore, after hydrogenation, liquid CaH_4_ became solid CaH_4_ phase, since the driving force, hydrogenation enthalpy, was no longer applied to the system. Fig. 3*E* shows the enthalpy changes at each stage of CaH_2_ hydrogenation via surface melting. The enthalpy of liquid CaH_4_, serving as an intermediate state, gradually decreases with increasing pressure, confirming how elevated pressures reduce the activation energy and enhance the hydrogenation reaction. This finding explains why we sometimes need to apply, in experiments, an “overpressure” that is higher than the equilibrium pressure predicted by DFT calculations.

## Discussion

In summary, the hydrogenation reaction for the polyhydride is thermodynamically understood from the enthalpy of the fusion for polyhydride products (Δ*H*_fus_), the hydrogenation reaction enthalpy (Δ*H*_hyd_), and the interfacial energies (γ_interface_). Specifically, polyhydride formation occurs via surface melting, reducing the activation energy required to obtain a liquid polyhydride phase as an intermediate state by controlling these physical properties with appropriate pressure and temperature. This understanding aligns well with the experimental observation that using BH_3_NH_3_ and a metal precursor is effective for polyhydride synthesis (9, 13). In this process, the metal precursor is not hydrogenated until BH_3_NH_3_ releases H_2_ molecules upon heating. This makes the enthalpy of the starting material (i.e., metal and H_2_ from BH_3_NH_3_) higher than that of a metal hydride and H_2_ molecules under high pressure. This further reduces the activation energy and promotes the formation of a liquid polyhydride phase as an intermediate state. As a result, surface melting is expected to occur even at lower temperatures than that for metal hydrides.

Owing to the thermodynamics of surface melting described above, theoretical structure search workflows are expected to provide substantial insights into experimental synthesis in the future. Specifically, after predicting a new polyhydride structure, one can i) calculate the pressure dependence of hydrogenation reaction enthalpy and ii) estimate the melting point of the product to evaluate its feasibility for subsequent experiments. Note that, in this study, the melting point is defined as the lowest temperature at which a given MH_x_ (M:H = 1:x) reaches equilibrium with either its liquid phase or a molten state composed of metal and hydrogen species (H or H_2_) with the same stoichiometric composition. While this definition remains consistent, if the molten state is stable, it is sufficient to replace ∆*G*_fus_ in Eq. **4** with the equilibrium between the solid and the molten state. Even if other stable compositions coexist or the liquid phase adopts various stoichiometric compositions as a molten state, the melting point can still serve as a practical indicator of the reaction temperature required to promote hydrogenation, as discussed in *SI Appendix*. However, it is critical to note that, particularly in the presence of molten states, precise control of both the composition and temperature of the system is necessary to improve the yield of the target material and to suppress the formation of amorphous structures.

Although constructing MLPs for interfacial reactions, as in this study, is time-consuming, workflows for bulk systems have already been established (56). Therefore, it is straightforward to develop MLPs for a homogeneous bulk system and estimate a rough melting temperature from the temperature-dependent enthalpy calculations. Consequently, we expect that the criteria derived from hydrogenation reactions via surface melting in our MLP-MD simulations, i) the pressure dependence of hydrogenation reaction enthalpies and ii) the melting temperature of polyhydride products, significantly accelerate the synthesis of new polyhydrides, integrating them with conventional computational techniques.

Building on the observed similarity to oxidation (50) and hydrogenation (48) reactions at ambient pressure, our findings suggest general principles for pressure-driven solid-state reactions. The surface melting mechanism, with its pressure-mediated activation energy, highlights a reaction pathway that may extend to other functional materials such as oxides and nitrides. While challenges remain in generating training data for complex, unknown phenomena, our application of MLPs to such a heterogeneous interfacial reaction demonstrates their emerging potential for predictive modeling in previously unexplored domains of materials science, laying the groundwork for predictive design of materials synthesis in practical applications.

## Materials and Methods

### Training Data Collection by DFT Calculation.

DFT calculations for training data collection were performed using the Vienna Ab initio Simulation Package (VASP) (57–59) with the Perdew–Burke–Ernzerhof (PBE) exchange–correlation functional (60). Standard PBE PAW potentials (61) were used to represent the core states, whereas the valence states (Ca 3p^6^4 s^2^, and H: 1 s^1^) were treated explicitly by the plane-wave basis-set with an energy cutoff of 400 eV. Only the Γ point was sampled for the Brillouin zone integration in most of the calculations listed in *SI Appendix*, Table S2. Note that we did not employ the high-pressure structure of CaH_2_ with *P6_3_/mmc* symmetry (62), but instead used the ambient-pressure structure with *Pnma* symmetry in these calculations. However, both structures share an hcp-like Ca sublattice. Therefore, the choice of the crystal structure for the training data does not significantly affect the results of the NPT-MD simulations under high-pressure conditions. DFT and ab initio MD (AIMD) simulations were carried out on the basis of the above calculation conditions for the systems listed in *SI Appendix*, Table S2.

### MLP Construction.

The MLP was trained using the Deep Potential-Smooth Edition scheme as implemented in the DeePMD-kit package (30, 63–65). We chose an embedding network with three hidden layers and (25, 50, 100) nodes per layer. The size of the embedding matrix has been set to 16. Three hidden layers with (240, 240, 240) nodes per layer were used in the fitting network. The cut-off radius was set to 6.0 Å and the descriptors decay smoothly from 0.35 to 6.0 Å. Approximately 58,000 DFT trajectories were employed as the training dataset as summarized in *SI Appendix*, Table S2. The mean absolute error (MAE) of atomic forces between DFT and MLP calculations was around 0.3 eV/Å for training and testing data below 3,000 K and 50 GPa (see *SI Appendix* for detailed discussion), which is comparable to those in previous studies (35, 38). In addition, local structures of the CaH_2_/H_2_ interface during the MLP-MD simulation described below were consistent with those of bulks, such as CaH_2_, CaH_4_, and H_2_, showing that these MD simulations were performed in the interpolated region of the constructed MLP or close to the interpolated region (*SI Appendix*, Figs. S9 and S10).

### MD Simulation.

The large-scale atomic/molecular massively parallel simulator [LAMMPS (66)] was used for MD simulations. A Nose–Hoover thermostat (67) and a barostat (68) were used for the NPT-MD simulations. The timestep was set to 1 fs and the mass of deuterium (*D*) was used instead of that of hydrogen. *SI Appendix*, Fig. S11 shows the initial coordinates used in the MLP-MD simulations in the main text. The CaH_2_/H_2_ interface containing about 2,000 atoms (Ca, 288 atoms; H, 1,728 atoms) was employed for obtaining the results in Figs. 1 and 2 owing to the computational cost of the inversion analysis of the PD, whereas those with 4,000 atoms (Ca, 576 atoms; H, 3,456 atoms) were used for obtaining the results in Fig. 3 and *SI Appendix*, Fig. S6. The cell parameters for the initial coordinates are 17.3 × 19.2 × 65.2 Å^3^ and 16.8 × 19.2 × 88.5 Å^3^, which are sufficiently large compared with the MLP cutoff radius (6 Å). The surface energy database by Tran et al. (69) demonstrates that a slab thickness of approximately 1 nm converges the surface energy to within an error margin of 0.02 J m^–2^. Based on this, the slab models used in this study, with thicknesses of several nanometers, ensure that the properties of the slab interior are representative of bulk behavior. For these interface systems, we started to conduct MLP-MD simulations at a low temperature and a low pressure of 300 K and 10 GPa, respectively, so that hydrogenation did not occur during the relaxation of the initial configuration. Then, the system was pressurized and heated for about 50 ps and maintained at a fixed temperature and pressure after 50 ps, as shown in the time series of temperature and pressure in Fig. 1*B*. Ovito (44, 70) was used for visualization and adaptive common neighbor analysis (a-CNA) of the MLP-MD simulations. Topological data analysis (TDA) based on persistent homology has been conducted using Homcloud (45) package. The detail of persistent homology analysis and subsequent analysis are described in *SI Appendix*. WulffPack (71) was used for the analysis of the Wulff shapes. To obtain Wulff shape for CaH_2_ nanoparticle, we analyzed the surface energy for (100), (010), (001), (012), and (103) surfaces. Then, the surface structure of CaH_2_ nanoparticle with 10,000 atoms was optimized to minimize the total surface energy. The surface energies for these surfaces were obtained using VASP and summarized in *SI Appendix*, Table S3.

## Supplementary Material

Appendix 01 (PDF)

Movie S1. Movie of atomic configurations during MLP-MD simulation for CaH_2_(100)/H_2_ interface at 1500K under 40 GPa.

## Data Availability

The data generated in this study have been deposited in the Zenodo (72) repository (73). All other data are included in the manuscript and/or supporting information.
