# Supplementary material for: Surface melting–driven hydrogen absorption for high-pressure polyhydride synthesis
Source: Proc Natl Acad Sci U S A. 2025 May 29;122(22):e2413480122. doi: 10.1073/pnas.2413480122 (PMC12146707; doi:10.1073/pnas.2413480122)
Supplement: Supplementary file 1 — Appendix 01 (PDF) [file pnas.2413480122.sapp.pdf]

PNAS

Supporting Information for

Surface Melting-Driven Hydrogen Absorption for High-Pressure Polyhydride Synthesis

Ryuhei Sato*, Lewis J. Conway, Di Zhang, Chris J. Pickard, Kazuto Akagi, Kartik Sau, Hao Li, Shin-ichi Orimo*

Ryuhei Sato (RS), Shin-ichi Orimo (SO)
Email: rsato@material.t.u-tokyo.ac.jp (RS), shin-ichi.orimo.a6@tohoku.ac.jp (SO)

This PDF file includes:

Supporting text
Figures S1 to S24
Tables S1 to S5
Legends for Movies S1
SI References

Other supporting materials for this manuscript include the following:

Movies S1

Supporting Information Text

Strategy of DFT training data collection

The DFT calculation data were collected on the basis of the following ideas. The purpose of this MLP is to reproduce the CaH_2 hydrogenation reaction. Therefore, multiple CaH_x compositions (Ca, CaH_2, CaH_4, CaH_6, and H_2) were trained to account for the compositional changes during the hydrogenation reaction. The transition state of the chemical reaction is usually less stable than the local minimum. Therefore, we sampled energetically unstable states by performing AIMD at very high temperatures (e.g., T = 3,000 and 10,000 K). Indeed, AIMD simulation for CaH_4 at 3000 K shows the decomposition reaction, as seen in the snapshot in Fig. S12. Such sampling of unstable states at high temperatures and AIMD simulations for mixtures of CaH_x + H_2 (x = 2, 4) (Fig. S13) contribute to the MLP accuracy of the CaH_x/H_2 interface and hydrogenation reactions reproduced in the MLP-MD simulations. To avoid the underestimation and overestimation of interatomic forces, structural optimizations were performed for systems with lattice constants compressed to 80% and expanded to 120% on the basis of the report by Shimamura et al. (***1***). The structural optimization results under various pressures from 0 to 150 GPa (for Ca, only ~50 GPa) were also added to the training dataset to reproduce pressure-dependent energy changes of each phase (Ca, CaH_2, CaH_4, CaH_6, and H_2).

Discussion on the accuracy of the constructed machine learning potential

Figure S14 shows the force on each atom obtained from MLP and DFT calculations, using atomic coordinates during AIMD simulations. Note that these figures show two-dimensional probability density distributions with 0.02×0.02 (eV/Å) meshes. The maximum value of a mesh in the probability density distribution (P/P_{max}) is normalized to be 1. As shown in Fig. S14, the MLP forces for the same atomic coordinates with AIMD simulations for the bulk systems (Ca, CaH_2, CaH_4, and H_2) are on y = x. This confirms that MLP can accurately represent the physical properties of these bulks. The calculated mean absolute error (MAE) is about 0.3 eV/Å below 3000 K and 50 GPa (Table S2). Figure S15 shows the atomic forces determined by MLP and DFT calculations using AIMD trajectories for the CaH_2/H_2 and CaH_4/H_2 interfaces (see Fig. S16 for initial and final configurations of these AIMD simulations). The results are also on y = x, even though they are not included in the training dataset for MLP construction. Therefore, although the error is slightly large (MAE ≤ 0.42 eV/Å), we conclude that the MLP captures the behavior of the H_2 molecule and CaH_x slab near the interface. This is further supported by force comparison between MLP and DFT calculations for atomic coordination during MLP-MD simulation for CaH_2 hydrogenation and CaH_4 dehydrogenation. Figure S17 shows the forces determined by MLP and DFT calculations using the atomic coordinates obtained in MLP-MD simulations. These MLP-MD simulations reproduced CaH_2 hydrogenation and CaH_4 dehydrogenation, as shown in the time series of H/M ratio in Fig. S18. The forces during these MLP-MD simulations are almost identical to those obtained from the single-step DFT calculations, and MAE between MLP and DFT is as small as 0.2 eV/Å. The reason why the MAE of these simulations (Fig. S17) is smaller than those for the interfaces in Fig. S15 is because in Figs. S14 and S15 were the atomic forces obtained from AIMD simulation trajectories, whereas that shown in Fig. S17 was calculated from the MLP-MD simulation trajectories. That is, the atomic forces shown in Fig. S17 is expected to have a lower MAE because these MLP-MD simulation trajectories include only atomic coordinates that can agree with the potential energy surface of MLP. On the other hand, the MAEs between MLP and DFT during hydrogenation and dehydrogenation in these MLP-MD simulations are small. This does not guarantee that the reaction paths during these MLP-MD simulations are minimum-energy paths. However, it is clear that these reaction paths were reproduced with atomic forces and energy equivalent to those obtained in DFT calculations. In this sense, it is guaranteed that the reaction paths obtained by MLP-MD simulations are physically valid ones. As shown in the pair distribution function (PDF), $g_{A\text{-}B}(r) = n_{A\text{-}B}(r)/(4\pi r^2 dr)$ in Fig. S9, the local structures around Ca during the hydrogenation reaction at the $CaH_2(100)/H_2$ interface at 1500 K under 40 GPa are smoothly shifted from that of CaH_2 to CaH_4. In addition, persistence diagrams (PDs) during the MLP-MD simulation in Fig. S10 show almost all the local ring structures during hydrogenation agrees with those of CaH_2, CaH_4, and H_2 bulks. These results also show that MLP-MD simulations in this study were performed in the interpolated region of the constructed MLP or close to the interpolated region.

The MAE of the constructed MLP is comparable to those in previous studies by considering high-pressure use of this MLP. For example, the root mean squared error (RMSE) = 0.2 eV/Å for single-component H_2(***2***) and RMSE ≈ 30 kcal/mol/Å (= 1.3 eV/Å) for the four-component system CHNO (***3***) were previously reported. The RMSEs tend to be larger than MAE. In addition, the MAE and RMSE should increase as the number of elements increases owing to the complexity of the sampling space. Considering these factors, the MAEs summarized in Table S2 in this study are reasonably accurate compared with the potentials for high-pressure simulations in previous studies. Note that these MAEs are larger than the MLPs designed for calculations at ambient pressure. However, as seen in the pressure-dependent MAE change for H_2-molecule AIMD simulation results in Fig. S19, MAE is proportional to the operation pressure, probably because high pressure increases the absolute value of atomic forces. Therefore, relatively large MAEs are inevitable in the MLPs for the calculation under high pressure. Note that the MLP-MD simulations in this paper were conducted below 50 GPa, suggesting that MAEs during these MLP-MD simulations are expected to be from 0.2 to 0.4 eV/Å on the basis of the values in Table S2. Also note that the MAE between MLP and DFT calculations for AIMD simulation trajectories for Ca and CaH_2 at P=0 GPa and 1000 K is smaller than 0.1 eV/Å, which is comparable to those previous studies at ambient pressure (***4***,***5***).

Topological data analysis (TDA) based on persistent homology

Persistent homology is a mathematical framework for analyzing n-dimensional holes in the given discrete data such as atomic coordinates. In materials science, it has been used for the structural analysis of materials such as amorphous and glass materials (***6***,***7***). More recently, there have been reports of its applications to dynamic processes such as ion transport and nucleation (***7,8***). Therefore, it is effective for the analysis of reaction processes involving disordering, such as surface melting in this study. Here, we focus only on one-dimensional pores (ring structures) defined on homology to analyze specific structures during hydrogenation. We employed a software package, Homcloud (***9***) for TDA based on persistent homology in this report. TDA based on persistent homology produces persistence diagrams as follows (see Fig. S20 for a schematic image),

1. Prepare atomic coordinates from MD simulation trajectories
2. For each atomic coordinate, place a fictitious sphere with the radius, r.
3. This radius r is gradually increased during persistence diagram making.
4. When the radius of this fictitious atomic spheres reaches a certain radius r_b, the sphere touches each other, making an empty space separated by the spheres. Here, r_b^2 is defined as birth, that is the x-axis in the persistence diagram.
5. As r is further increased, such an empty space is filled by the fictitious atomic sphere. The y-axis, that is, death, in the persistence diagram equals the power of the corresponding radius r_d^2.

The two-dimensional distribution of birth and death for the ring structures reflecting their size and plotting its frequency is the persistent diagram in this report. The birth value is proportional to the maximum distance between atoms in the ring structure, whereas “death – birth” is roughly proportional to the area of the empty space separated by the atomic ring. Therefore, the birth values of ring structures originating from the CaH_2 and CaH_4 structures in Fig. 2 are comparable to $(r/2)^2$ of the 1st and 2nd peaks in PDF for H atoms around the H atom in Fig. S21, respectively. Note that birth value is the bottleneck distance to form the ring structure, whereas PDF is the distribution of distance between atoms in all possible pairs. This means that the 1st peak of the PDF alone does not necessarily explain the birth of the persistence diagram.

For the inverse analysis, the stable volume method (***10***) was used. Specifically, the following operations were performed on the ring structures selected by this method and statistically analyzed.

i. Statistics were taken on the number of atoms constituting rings with the same (b, d), and the ring structure with the highest frequency of the number of atoms was selected as the representative ring structure for the corresponding (b, d).
ii. The Cartesian coordinates of each atom in these ring structures were extracted. The coordinates were recentered with the center of gravity as the origin. Then they are rotated so that the distance between the x–y two-dimensional plane and each atom was as small as possible.
iii. The normalized ring structures with the same (b, d) and number of atoms were overlapped to obtain an average ring structure. Specifically, these ring structures were rotated around the z-axis. By this rotation, the average ring structure was obtained by overlapping and averaging the two rings so that the distance between the vertex atoms of the two rings was minimized.

Structure Prediction of Particular Composition

Universal Structure Predictor: Evolutionary Xtallography (USPEX) (***11-13***) was employed to predict the stable structure of particular compositions (e.g. $CaH_{3.5}$ and CaH_{24}). The population size of each generation in the genetic algorism was 20. The initial structures were created using random symmetric (***13***) and topological structure generators (***14***) with formular unit (*z*) = 1-4, while the subsequent generations contained 20% of random structures, and the remaining 80% of structures were created using the heredity and softmutation operators. Here, the evolutionary searches were combined with structure relaxations using DFT(VASP) calculations. The plane-wave energy cut off was set to 450 eV. Note that the pressures during the structure search for $CaH_{3.5}$ and CaH_{24} were 50 and 150 GPa, respectively. Figure S22 shows the snapshots of the most stable structures among these calculations. The atomic configurations (POSCAR format) are summarized in Table S4 and S5.

Liquid phase vs Molten state in our MLP model

To determine whether CaH_4(l) or a molten state is formed, we performed 0.5-ns NPT-MLP-MD simulations on the CaH_4/H_2 interface at temperatures ranging from 1500 K to 4000 K under 40 GPa. Our MLP model successfully reproduced both the liquid phase of CaH_4 and the molten state (a mixture of Ca, H, and H_2). Snapshots from the simulations are shown in Fig. S4. Starting from the MD results for the CaH_2(100)/H_2 interface at 40 GPa and 1500 K (Fig. S4A), we observed the following:

(1) The boundary between H_2(l) and CaH_4(l) disappears at 2800 K, showing the formation of a molten state (Fig. S4B).
(2) This boundary remains distinct at 2400 K (Fig. S4C), confirming that the transition from CaH_4(l) to the molten state occurs between 2400 K and 2800 K.

Additionally, the simulations show that CaH_4(s) melts and loses structural order between 1600 K and 1800 K, placing the melting point within this range. As shown in Figs. S4D and S4E, the hydrogen atom number density between the H_2 and CaH_4 phases remains nearly constant before and after melting, confirming a composition close to CaH_4 and H_2. These results distinguish the equilibria CaH_4(s) + H_2(l) ↔ CaH_4(l) + H_2(l) ↔ molten state (Ca + H + H_2, M:H = 1:4), supporting the validity of the proposed surface melting reaction model. Furthermore, the solubility of Ca in H_2(l) is negligible below 2400 K but increases abruptly near the transition from the liquid phase to the molten state.

The Effect of variational composition on the melting-point criterion

To examine the effect of variational composition on the melting-point criterion, we discuss the phase diagram of the Ca-H system based on our MLP results and prior studies. In this study, the melting point (T_m) is defined as the lowest temperature at which a given MH_x (M : H = 1 : x) reaches equilibrium with either its liquid phase or a molten state composed of the metal and hydrogen species (H or H_2) with the same stoichiometric composition. The primary objective is to elucidate the hydrogenation reactions rather than to construct a precise phase diagram. Therefore, to simplify the analysis, the following assumptions are made:

1. Sufficient Hydrogen Supply: The system has an abundant hydrogen source, ensuring that the H/M ratio exceeds that in target polyhydride, MH_x, (i.e., H/M > x).
2. Stable Phases Only: Only known stable phases are considered, excluding potential high-temperature phases as a first approximation.

At approximately 40 GPa, CaH_2, $CaH_{2.5}$, CaH_4, and H_2 are stably present. Here, we focus on the synthesis of CaH_4 and construct a phase diagram for H/Ca > 4. The formation enthalpy of $CaH_{3.5}$ obtained from USPEX ($CaH_2 + H_2 \leftrightarrow CaH_{3.5}$) was +0.80 eV/unit, indicating instability. Similarly, CaH_5 has been reported as unstable (***15***). When considering potential hydrogen defects (δ) or interstitial hydrogen in $CaH_{4+\delta}$, δ is expected to fall within the range $-0.5 < \delta < 1$, limiting defects up to 2–3 mol%. Consequently, bulk non-stoichiometry was neglected in the phase diagram as a first approximation, and the solidus line was assumed to have negligible impact on the phase boundary. For the liquidus line, the formation enthalpy of CaH_{24} ($CaH_4 + 10\ H_2 \leftrightarrow CaH_{24}$) was +6.8 eV/unit at 40 GPa, indicating low Ca solubility in H_2(l) (< 3%) at 0 K. Furthermore, MLP-MD simulations under 40 GPa show no dissolution of Ca in H_2(l) below 2400 K. However, a molten state forms above 2800 K (Fig. S4), suggesting that changes in the liquidus line are negligible below 2400 K but become significant near the transition temperature to the molten state. Note that CaH_4(s) melts at around 1800 K during the MLP-MD simulation under 40 GPa, suggesting that the melting point is around this temperature. Thus, there is a wide gap between melting point and transition temperature to the molten state at 40 GPa,

Based on the above MLP-MD simulations and previous research, the phase diagram was deduced and shown in Fig. S23. Here, no higher-coordinate polyhydrides beyond CaH_4 exist, leading to an equilibrium between CaH_4 and H_2(l) in the broad range of this phase diagram. Therefore, from the perspective of surface melting and hydrogenation reactions, the equilibrium temperature (T_m) of CaH_4(s) ↔ CaH_4(l) serves as a critical criterion for the synthesis of CaH_4.

Discussion on the Molten State with Hypothetical Phase Diagrams

At ultra-high pressures, molten states may form instead of distinct liquid phases. C. M. Tenney et al. (***16***) reported a hydrogen phase diagram, showing that H_2(l) decomposes into a mixed liquid of H and H_2 at around 1000 K under 150 GPa. This indicates that molten states are more likely to form under such conditions, potentially exhibiting significant compositional variability. To provide additional insights, we constructed hypothetical phase diagrams (Fig. S24) using the deduced one in Fig. S23 based on the following assumptions:

(1) CaH_4 directly forms the molten state.

(2) CaH_6 is stable at the same pressure with both CaH_4 and CaH_6 forming only molten states.

As the Ca solubility increases with temperature, the liquidus line for H_2(l) (or molten state) converges toward the melting point of the stable compound (e.g., CaH_4) as the hydrogen mole fraction decreases (Fig. S24A). Therefore, the melting point of CaH_4 remains a critical upper limit for the hydrogenation reaction, even when molten states with variable compositions are present. If multiple stable compounds exist (e.g., CaH_4 and CaH_6), their respective melting points can determine the reaction temperature range. To maximize the yield of the target material, it is essential to carefully adjust the experimental stoichiometry to match that of the target compound. In this regard, the melting point of the target material serves as a valuable reference.

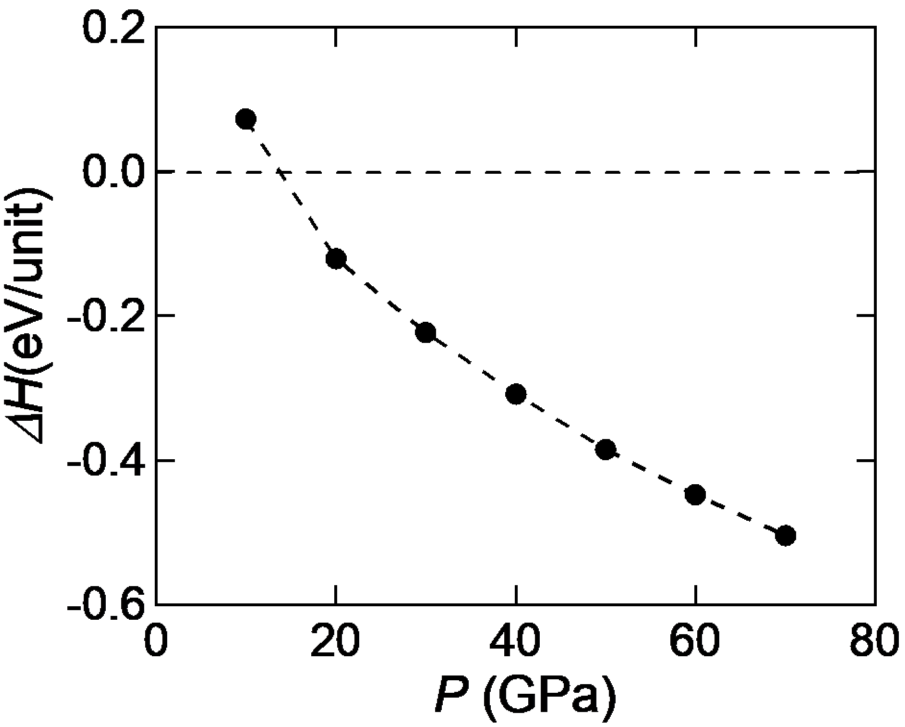

Fig. S1. The CaH_4 formation (CaH_2(s) + H_2(l) ↔ CaH_4(s)) enthalpy per unit as a function of pressure obtained from DFT(VASP) calculations. Here, the optimized energies and structures of CaH_2(*Pnma*), CaH_4, and H_2(*C2/c*) phases with 10×10×10 *k*-points were employed for this calculation.

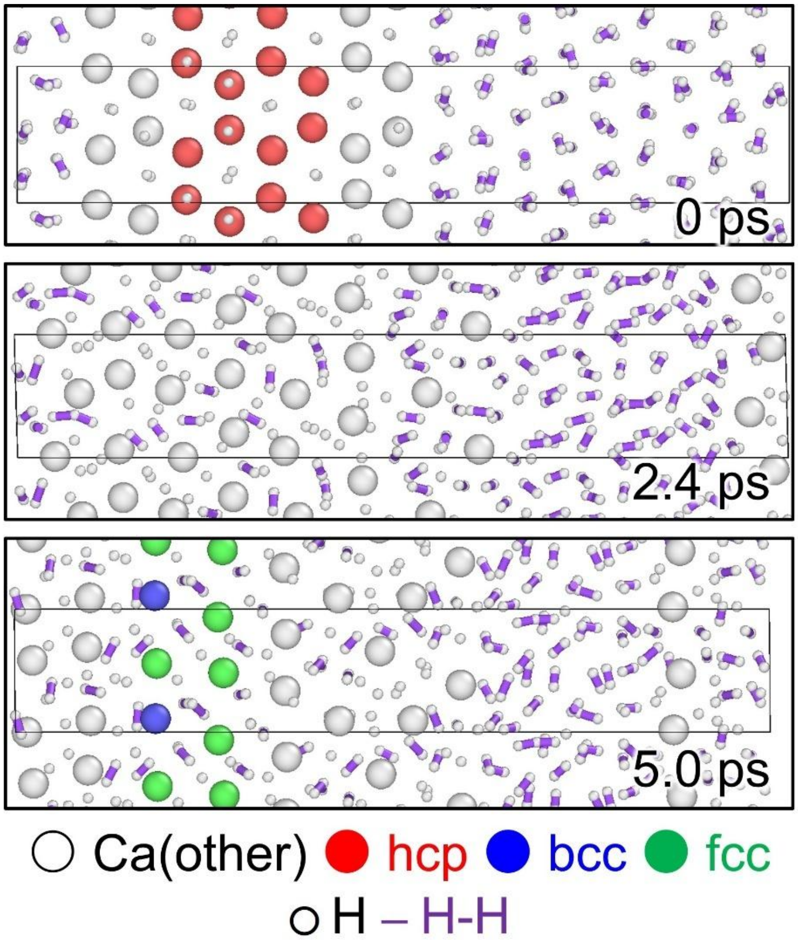

Fig. S2. Snapshots of CaH_2(100)/H_2 interface during 5-ps AIMD simulation at 50 GPa and 2000 K. Due to its limited size (16 Ca and 160 H atoms), the simulation is strongly affected by the periodic boundary condition. Nevertheless, significant rearrangement of Ca atoms was observed during hydrogen absorption. 2×2×2 *k*-point was employed for this calculation. Note that this AIMD simulation was not included in the training dataset for MLP construction.

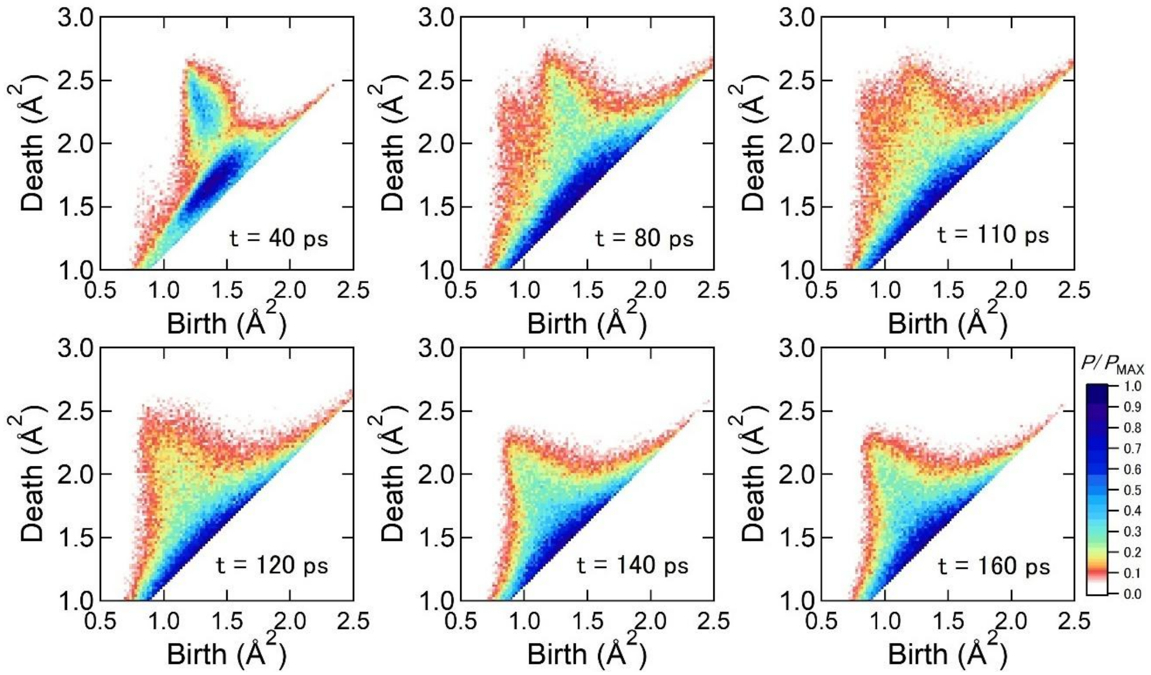

Fig. S3. Time-averaged persistence diagram only using H atoms near CaH_x bulk during the MLP-MD simulation for the CaH_2(100)/H_2 interface at 1500 K under 40 GPa. These persistence diagrams were created only with H atoms whose distance to the nearest Ca is shorter than 3 Å. Note that these diagrams are the time-averaged ones every 10 ps during MLP-MD simulation unlike those using all the trajectory in Fig. 2A and B.

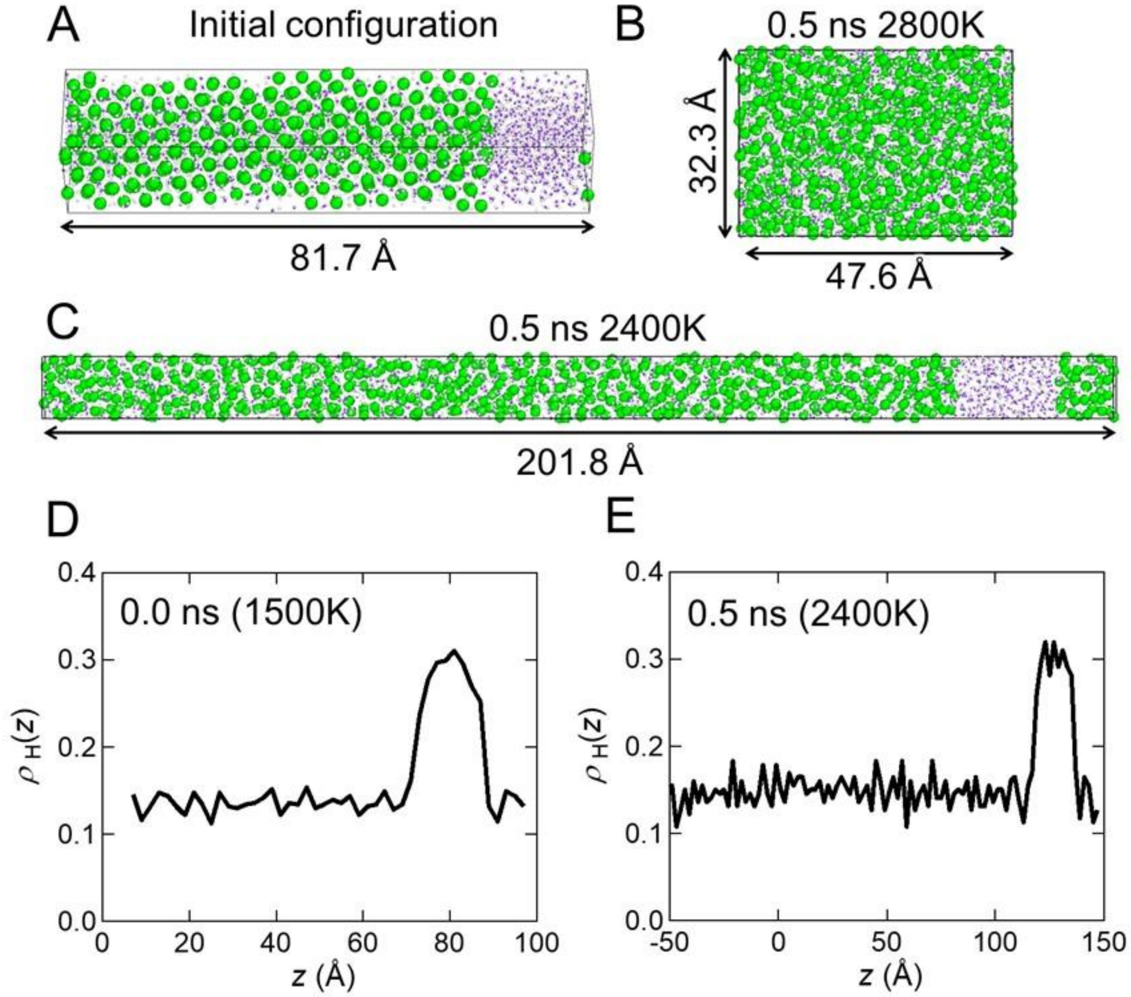

Fig. S4. Snapshots of MLP-MD simulations for the CaH_4/H_2 interface. (A): Initial configuration. (B): Molten state at 2800 K. (C): $CaH_4(l) + H_2(l)$ phase at 2400 K. Note that the system is significantly elongated to reduce interfacial energy at 2400 K. The CaH_4/H_2 interface was obtained by the hydrogenation reaction of $CaH_2(100)/H_2$ (with 576 Ca and 3456 H atoms) after 1-ns MLP-MD simulation at 40 GPa and 1500 K (Fig. 3). (D) and (E): Hydrogen atom density distribution along the z-axis (perpendicular to the interface) at 0 ns and 0.5 ns, respectively.

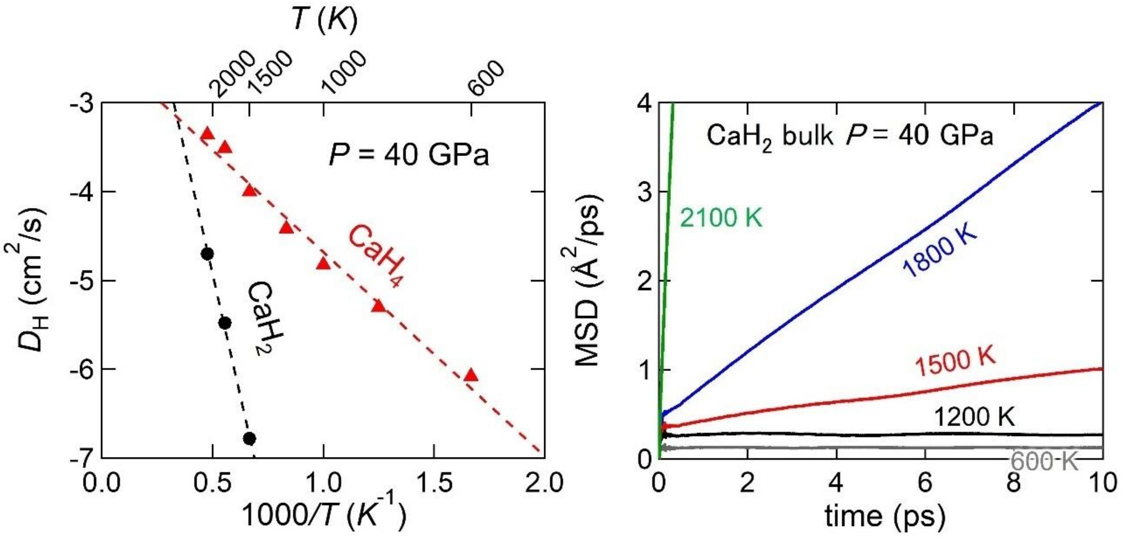

Fig. S5. (left) H diffusivity Arrhenius plot of CaH_2 and CaH_4 under 40 GPa (right) mean squared displacement (MSD) of H atoms during MLP-MD simulations of CaH_2 under 40 GPa. These data were taken from 100-ps MLP-MD simulations for 6×4×4 CaH_2 and 6×6×3 CaH_4 supercells.

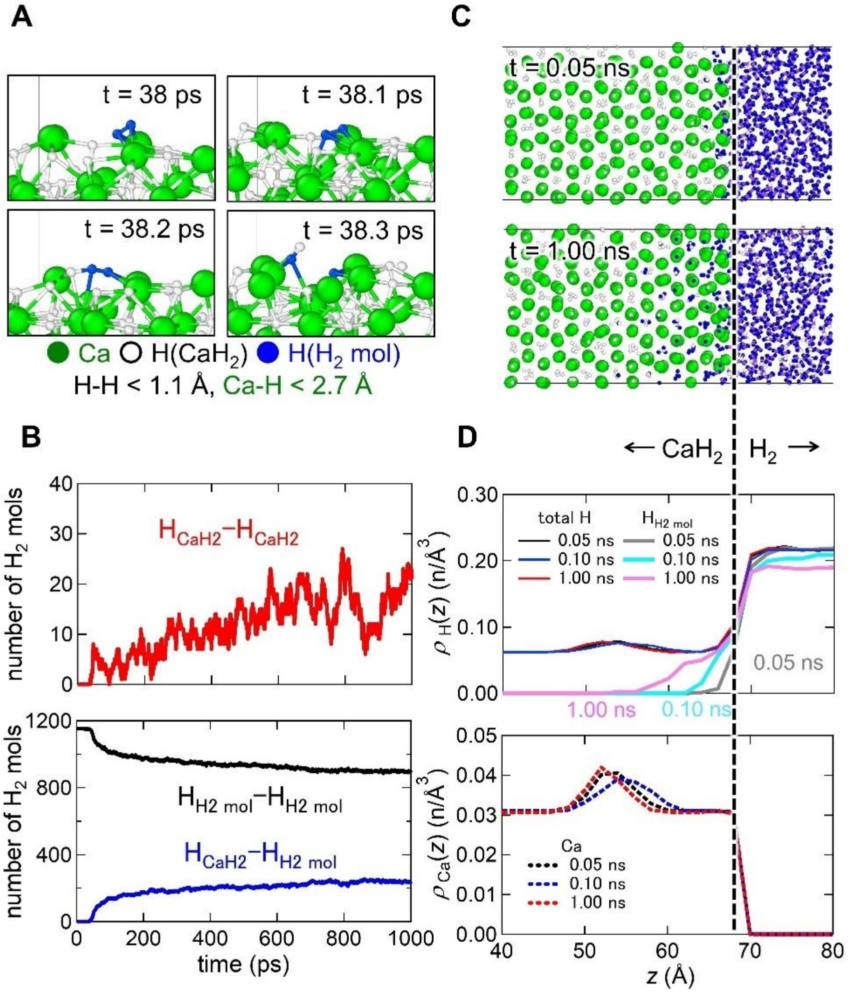

Fig. S6. (A) Snapshots of atomic configurations for H_2 dissociation on CaH_2 (100) surface (other H_2 molecules are not shown for clarity) and (B) time series of H_2 molecule numbers during MLP-MD simulation for the CaH_2(100)/H_2 interface at 1200 K under 15 GPa. Here, H atoms that were included in CaH_2 in the initial configuration (t = 0 ps) are denoted as H_{CaH2} and those forming H_2 molecules are denoted as $H_{H2\ mol}$. Then, H_2 molecules are classified into three groups: (red) H_2 molecules formed by two H_{CaH2} (blue) those formed by H_{CaH2} and $H_{H2\ mol}$, and (black) those that did not exchange H with CaH_2. H_2 molecules formed by H_{CaH2} gradually increases with time owing to H_2 dissociation and the subsequent H exchange. (C) Snapshots of atomic configurations for CaH_2/H_2 interface during MLP-MD simulation at 1200 K under 15 GPa. Green, white, and blue spheres represent Ca, H_{CaH2} and $H_{H2\ mol}$, respectively. (D) Time averaged atomic density as a function of z-axis perpendicular to the CaH_2(100)/H_2 interface. Each line represents the 5-ps average before the corresponding time. Here, the density distribution of all H atoms remains almost constant after 1-ns MD simulation. On the other hand, $H_{H2\ mol}$ distribution increased in CaH_2 bulk with time, showing that $H_{H2\ mol}$ diffusion from surface to CaH_2 bulk (i.e., H exchange between CaH_2 and H_2 molecules) occurred but did not contribute to H absorption to form CaH_4 without surface melting.

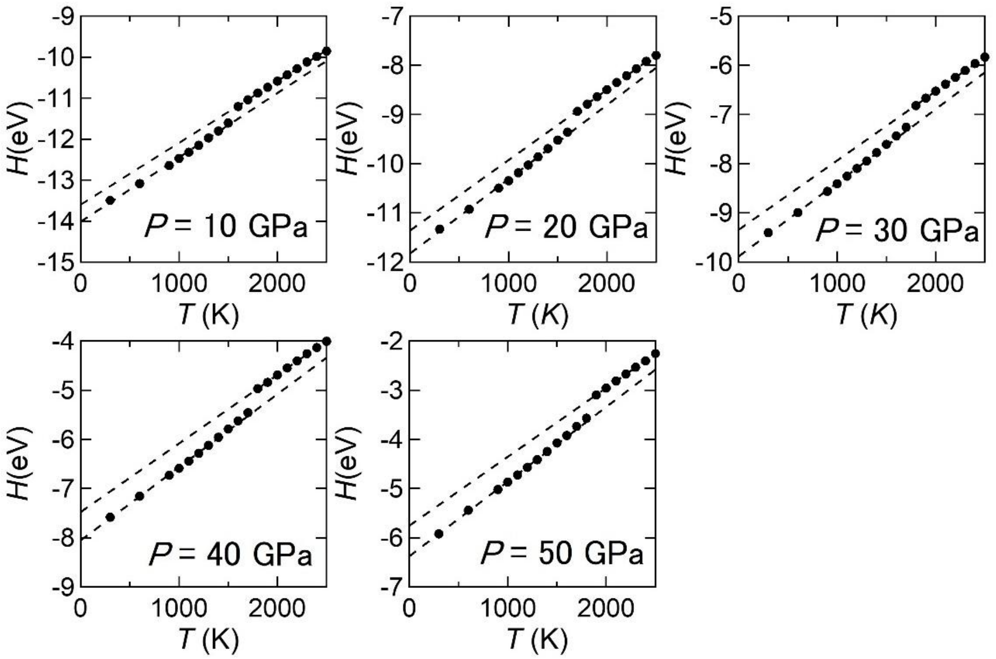

Fig. S7. Time averaged enthalpy during 100-ps MLP-MD simulation for 6×6×3 CaH_4 supercell under fixed pressure (10, 20, 30, 40, 50 GPa) and temperature (300–2500 K).

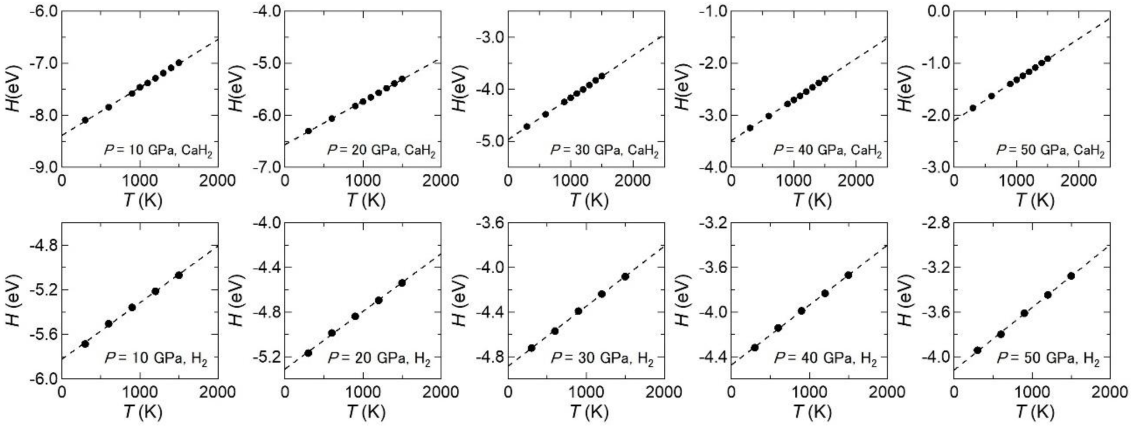

Fig. S8. Time averaged enthalpy during 100-ps MLP-MD simulation for (top) 6×4×4 CaH_2 and (bottom) 4×4×4 H_2 supercell under fixed pressure (10, 20, 30, 40, 50 GPa) and temperature (300–1500 K).

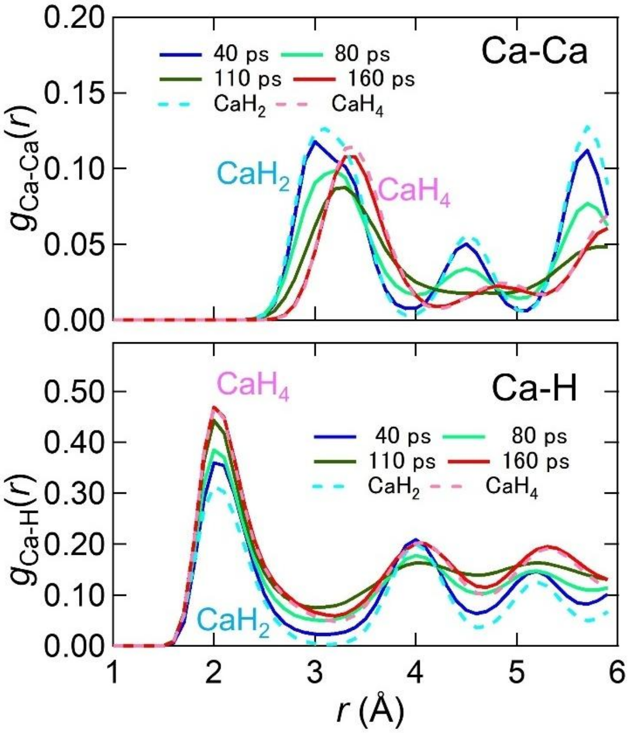

Fig. S9. Pair distribution function (PDF) of Ca and H atoms around Ca atoms during the MLP-MD simulation for the CaH_2(100)/H_2 interface at 1500 K under 40 GPa to reproduce hydrogenation reaction. The dashed lines represent PDF for the CaH_2 (blue) and CaH_4 (magenta) bulks during 100-ps MLP-MD simulations at 1500 K under 40 GPa. The cutoff radius for H/M ratio and persistence diagram for H atoms in Fig. 1 and 2 was chosen on the basis of the first peak in this PDF.

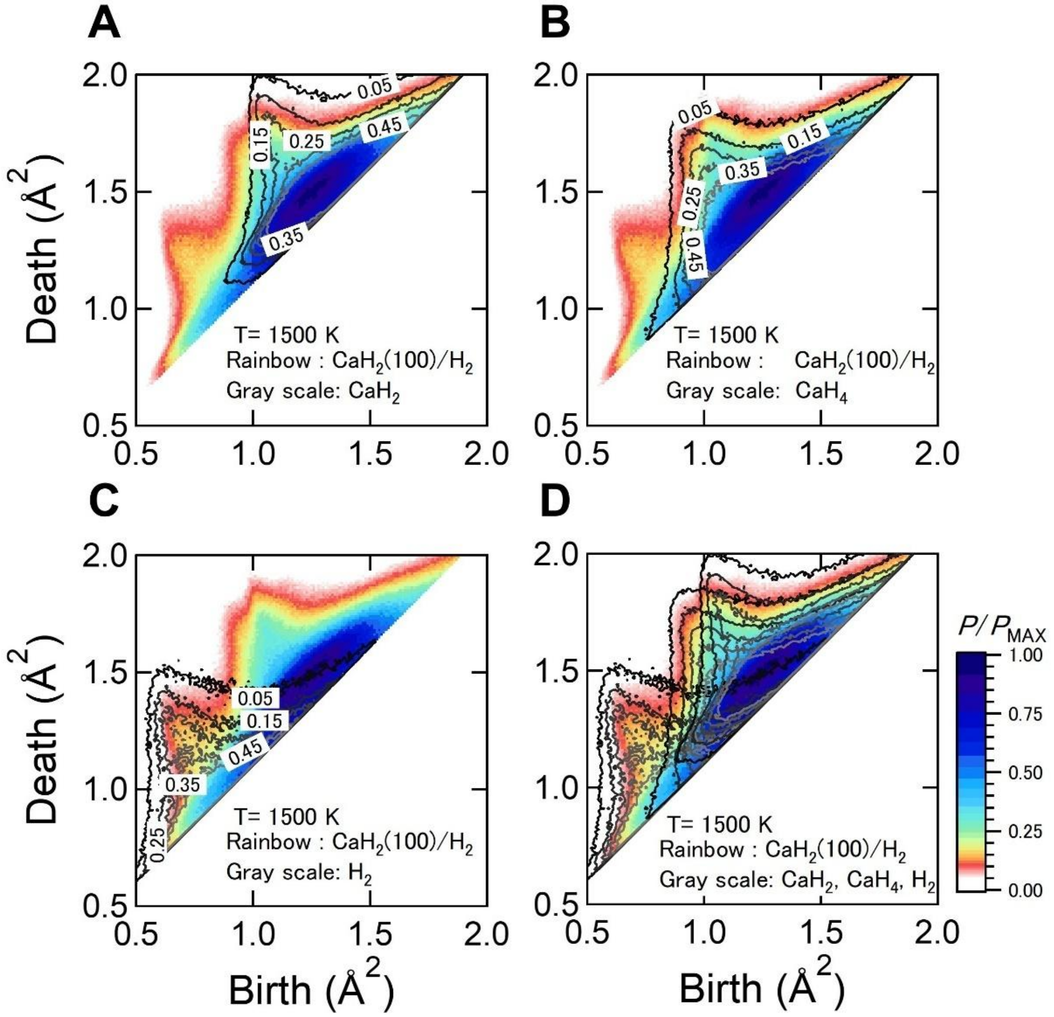

Fig. S10. Time-averaged persistence diagram for all Ca and H atoms during MLP-MD simulations of the $CaH_2(100)/H_2$ interface at 1500 K under 40 GPa from 25 ps to 160 ps (Rainbow color density plots). Gray-scale contour plots show PDs of 100-ps MLP-MD simulations at 1500 K for (A) CaH_2, (B) CaH_4, (C) H_2, (D) CaH_2 + CaH_4 + H_2 bulks. The PD of MLP-MD simulations of the $CaH_2(100)/H_2$ interface can be expressed by the overlapping of PDs for MLP-MD simulations of the CaH_2, CaH_4, and H_2 bulks.

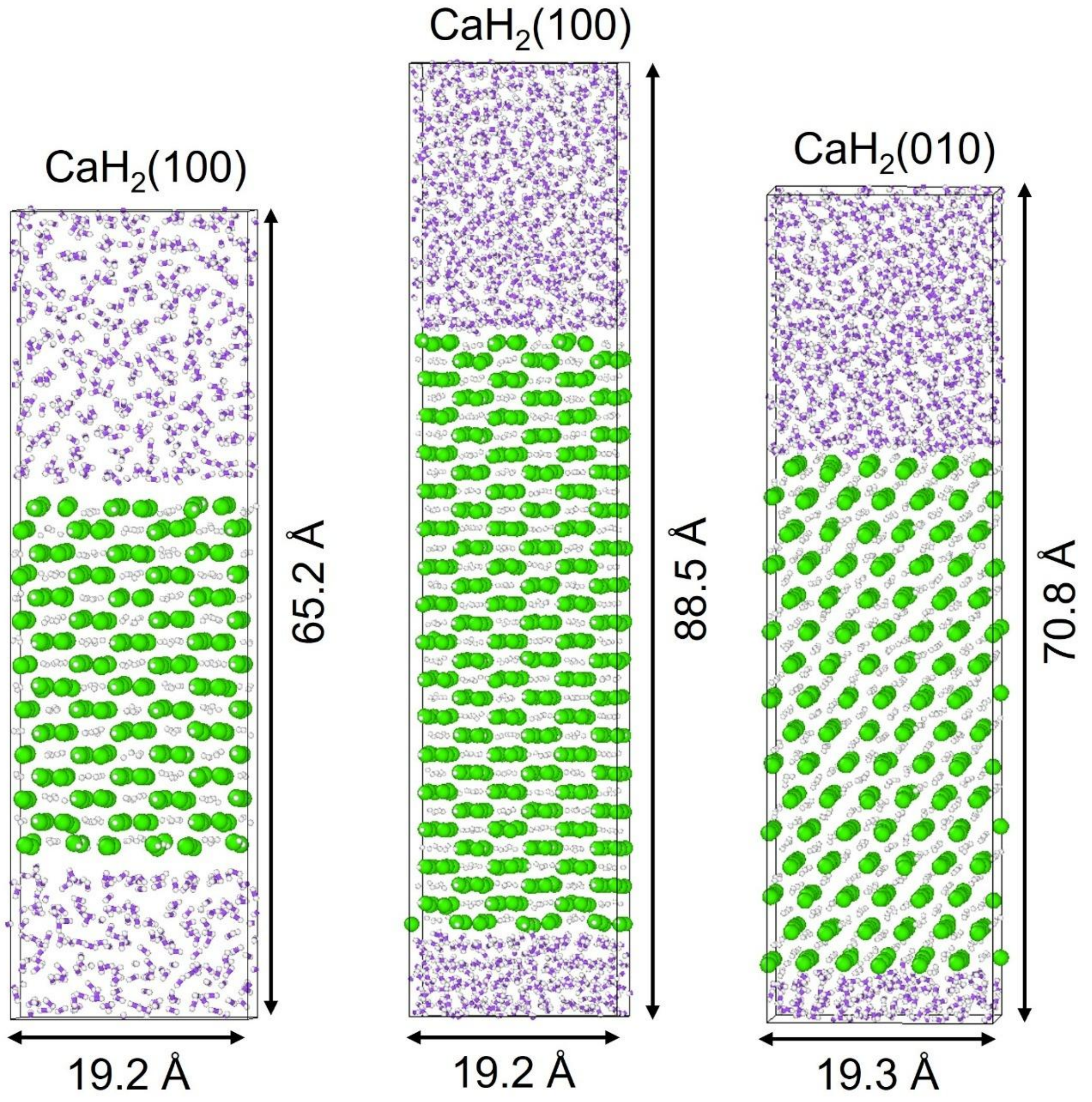

Fig. S11. Initial coordinates used for MLP-MD simulations of the hydrogenation reaction at the CaH_2/H_2 interface. (left) Coordinates with about 2000 atoms (Ca, 288 atoms; H, 1728 atoms) used for detailed analysis of the hydrogenation reaction (Figs. 1 and 2), including persistence diagram analysis; (center) and (right) coordinates with about 4000 atoms (Ca, 576 atoms; H, 3456 atoms) used to take pressure dependence of the hydrogenation reaction (Figs. 3A and 3B).

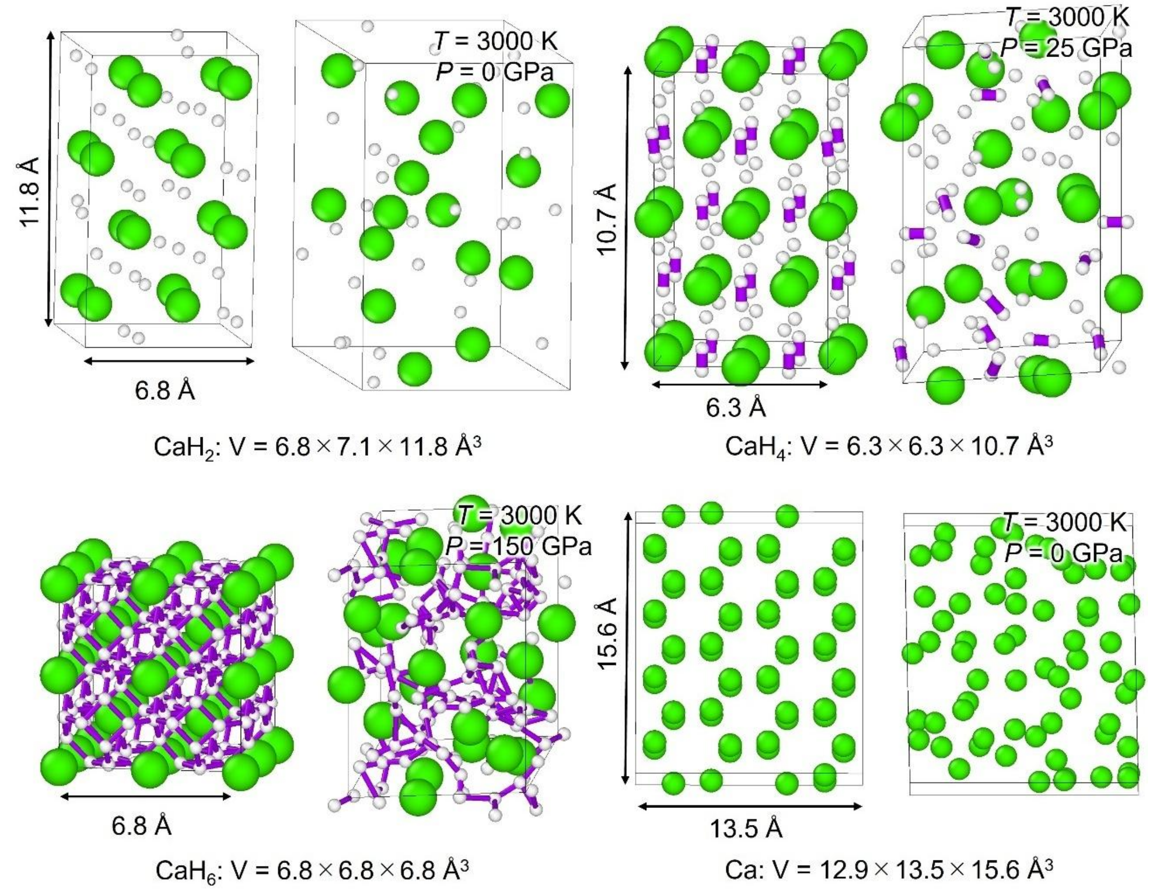

Fig. S12. Snapshots of the initial structures for CaH_2, CaH_4, CaH_6, and Ca bulks used for AIMD simulations and the final ones after AIMD simulations at 3000 K. The green and white spheres show Ca and H atoms, respectively. H–H bonds (CaH_4: < 1 Å, CaH_6: < 1.5 Å) were represented by purple lines. Cell parameters were selected so that the initial coordinates are larger than the MLP cutoff radius of 6 Å.

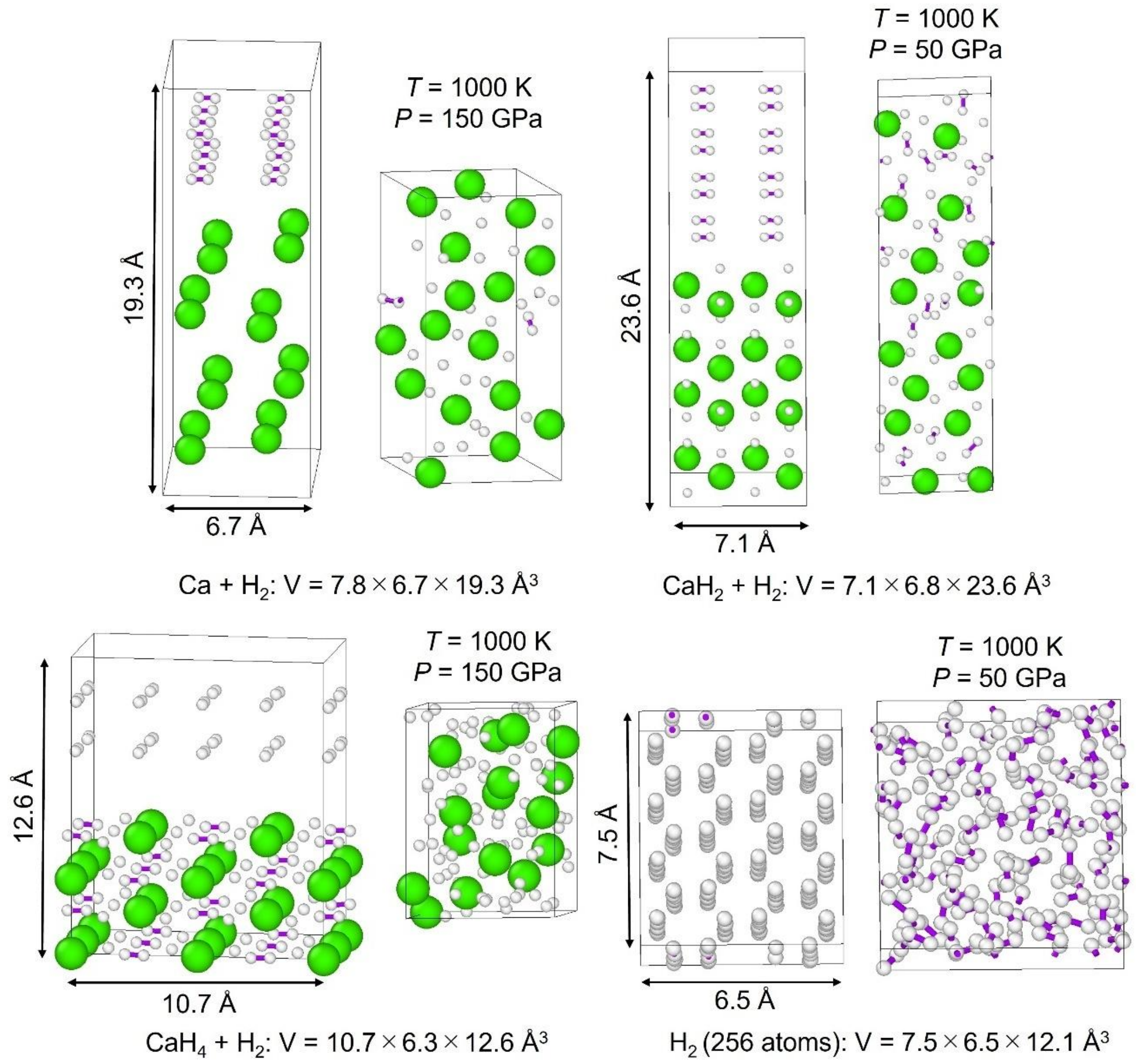

Fig. S13. Snapshots of the initial structure for the mixture of CaH_x and H_2 molecules or H_2 molecule system used for AIMD simulations and the final ones after AIMD simulations at 1000 K. The green and white spheres show Ca and H atoms, respectively. H–H bonds (< 1 Å) were represented by purple lines. Cell parameters were selected so that the initial coordinates are larger than the MLP cutoff radius of 6 Å.

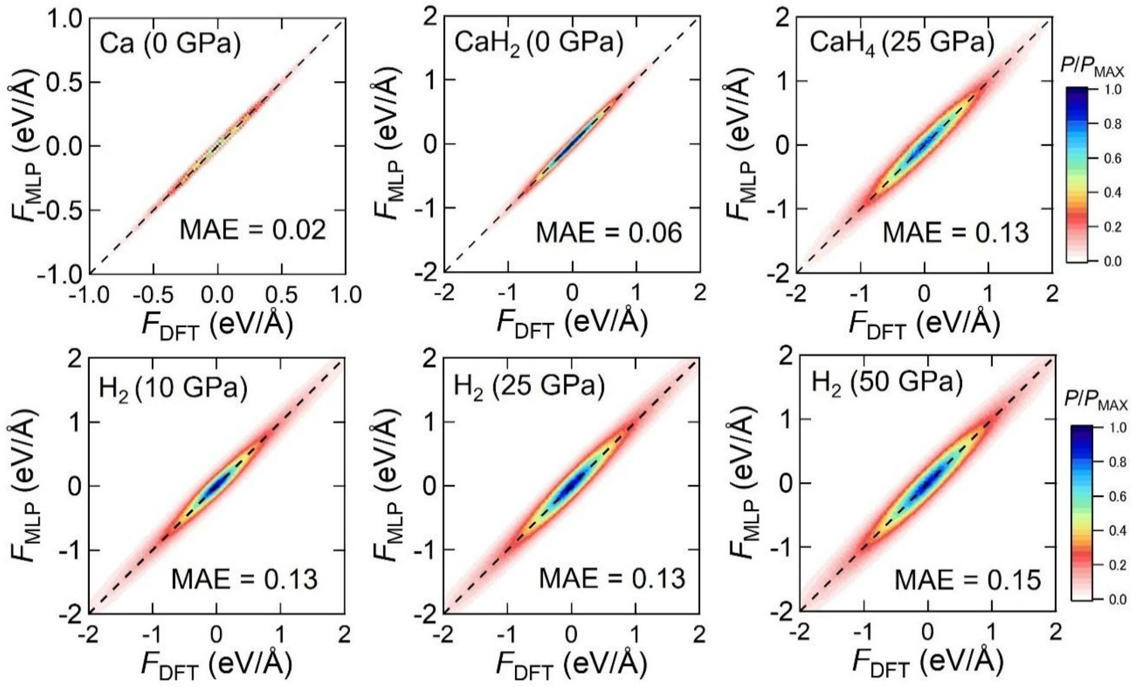

Fig. S14. Probability distribution plots of atomic forces for bulk systems (Ca, CaH_2, CaH_4, and H_2) obtained from MLP and DFT calculation. Here, all the trajectories during AIMD simulations of the corresponding systems were used to obtain these plots (see Table S2 for more detail for AIMD simulation condition). 0.02×0.02 (eV/Å) mesh was employed and the distributions were normalized using the maximum probability (P_{max}) in these probability density plots.

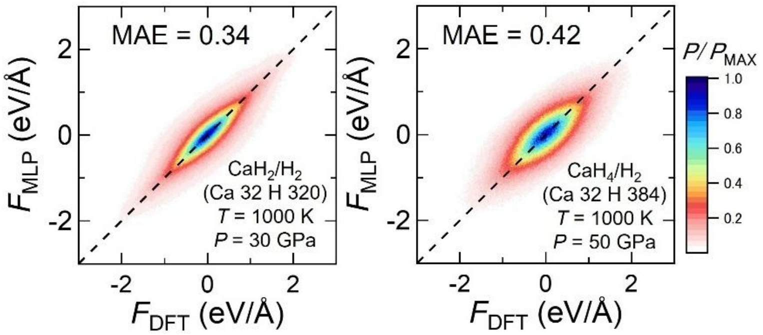

Fig. S15. Probability distribution plots of atomic forces for (left) the CaH_2/H_2 (Ca, 32 atoms; H, 320 atoms) and (right) CaH_4/H_2 (Ca, 32 atoms; H, 384 atoms) interface systems obtained from MLP and DFT calculations. Here, all the trajectories during AIMD simulations of corresponding systems were used to obtain these plots (see Table S2 for more detail). 0.02×0.02 (eV/Å) mesh was employed and the distributions were normalized using the maximum probability (P_{max}) in these probability density plots.

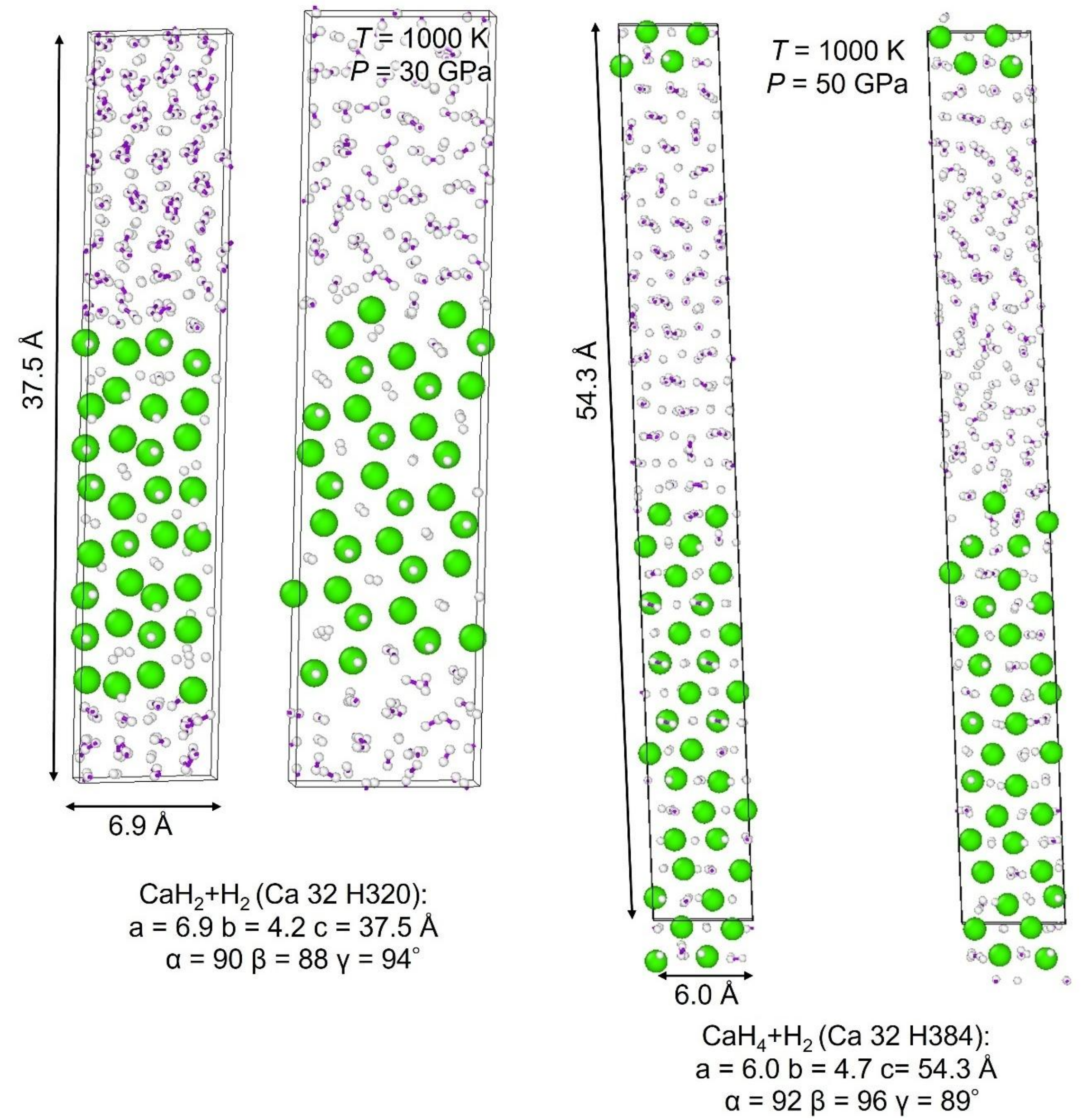

Fig. S16. Snapshots of the initial structure for the CaH_2/H_2 and CaH_4/H_2 interfaces used for AIMD simulations and the final ones after AIMD simulations at 1000 K. The green and white spheres show Ca and H atoms, respectively. H–H bonds (< 1 Å) were represented by purple lines. Note that these AIMD simulations were not used as the training dataset for MLP construction but used for the validation of MLP.

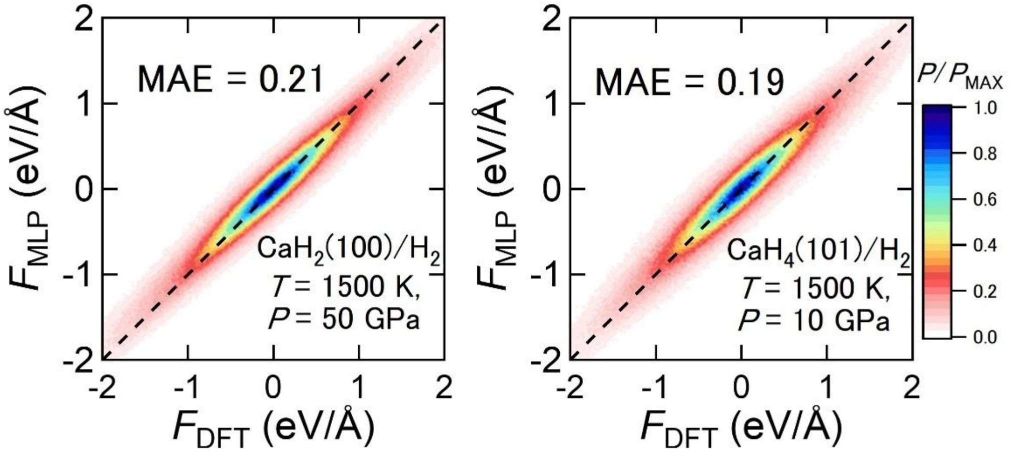

Fig. S17. Probability distribution plots of MLP and DFT atomic forces for MLP-MD trajectories during CaH_2 hydrogenation and CaH_4 dehydrogenation. Here, atomic configurations during MLP-MD simulation (summarized in Fig. S18) were taken every 0.1 ps and single-step DFT calculations were conducted to evaluate atomic forces. 0.02×0.02 eV/Å mesh was employed and the distributions were normalized using the maximum probability (P_{max}) in these probabilities density plots.

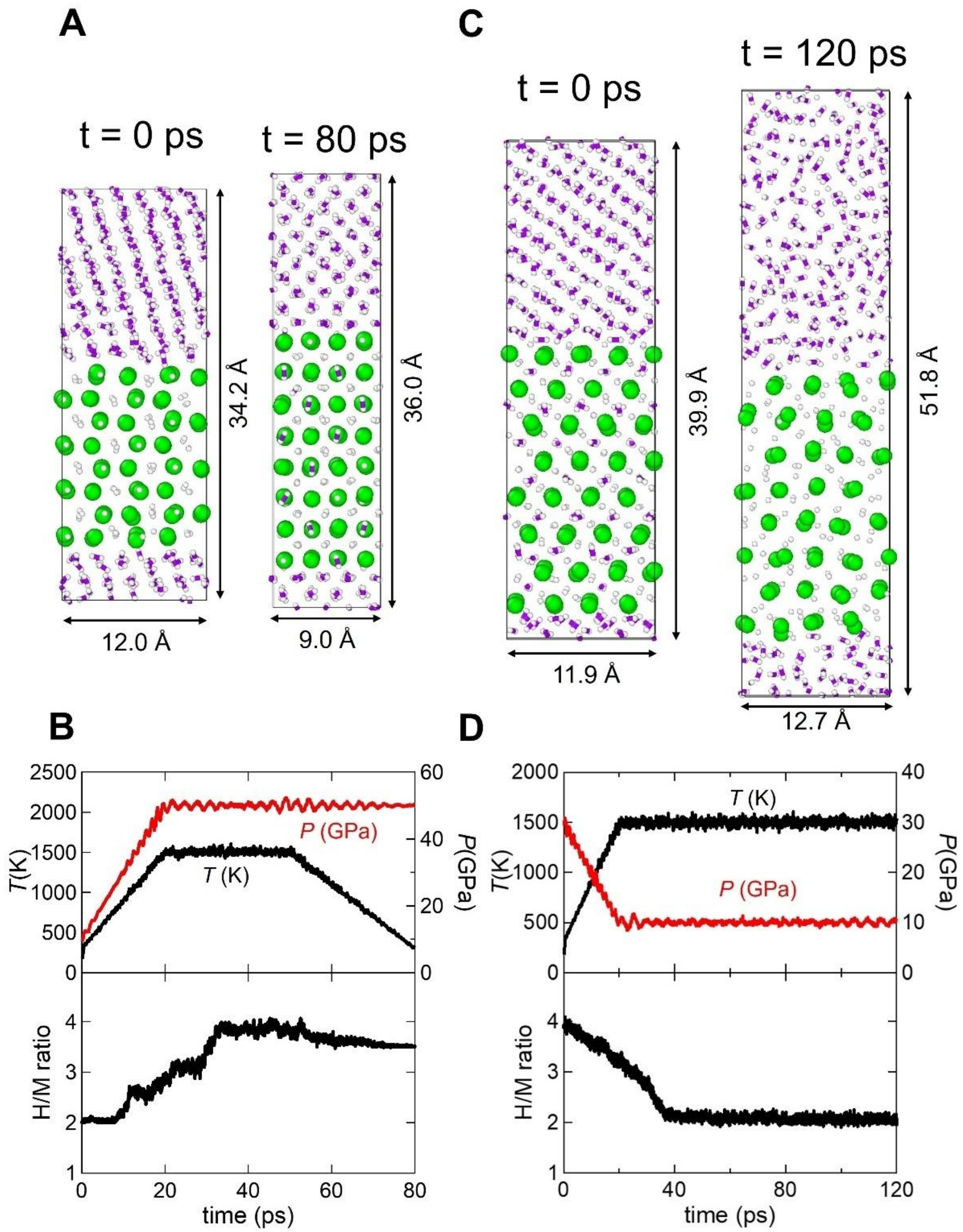

Fig. S18. Overview of MLP-MD simulations with a small simulation cell performed to compare MLP and DFT atomic forces. (A) Snapshots of initial and final coordinates and (B) time series of temperature, pressure, and H/M ratio during the MLP-MD simulation for CaH_2(100)/H_2 interface at 1500 K under 50 GPa for CaH_2 hydrogenation reaction. Note that after 20-ps quenching, CaH_4 contains H defects and H/M became 3.5, probably due to its relatively small systems size. In fact, H/M was 3.8 for the larger cell with 2000 atoms in Fig. 1, showing the strong size effect of this reaction. (C) Snapshots of initial and final coordinates and (D) time series of temperature, pressure, and H/M ratio during the MLP-MD simulation for the CaH_4(101)/H_2 interface at 1500 K under 10 GPa for CaH_4 dehydrogenation.

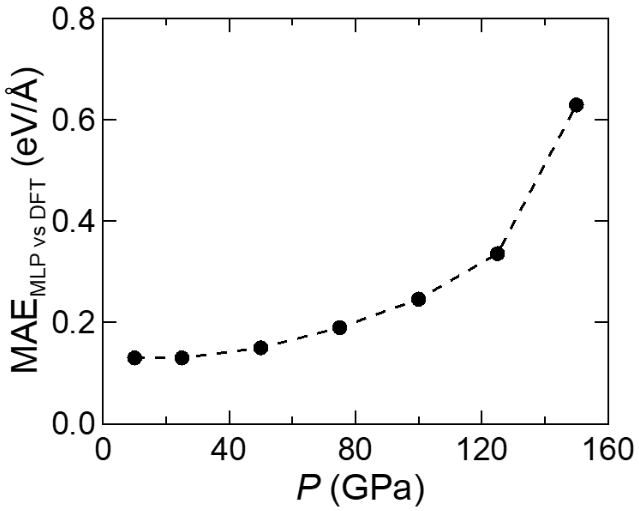

Fig. S19. Pressure dependence of mean absolute error (MAE) between MLP and DFT interatomic forces on atomic coordinates during AIMD simulations for 128 H_2 molecules at 1000 K.

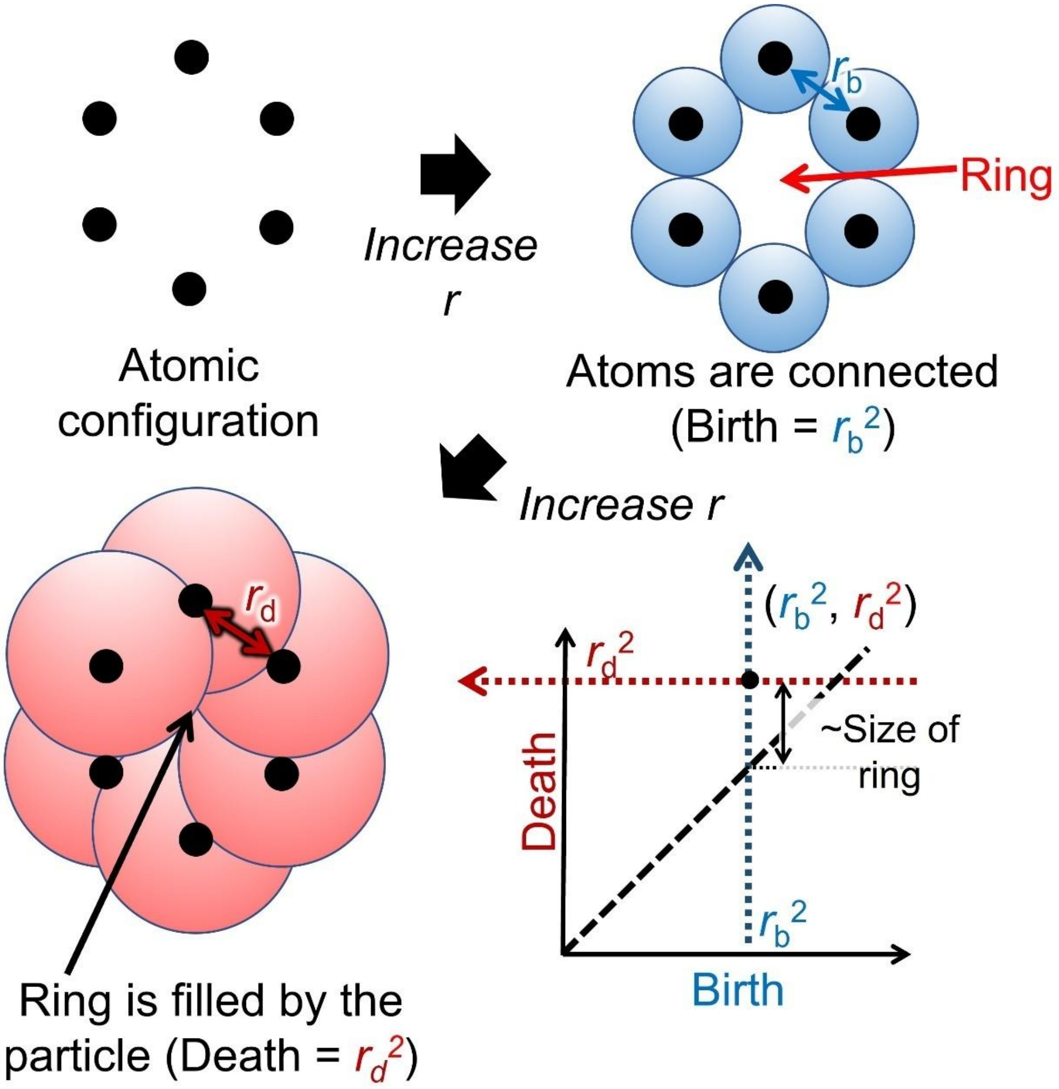

Fig. S20. Schematic diagram of how ring structures in an atomic configuration are analyzed by topological data analysis (TDA) based on persistent homology. In this analysis, we place a sphere with the same radius, *r*, at each atomic coordinate in the system. By increasing *r*, some atoms are connected to each other to form the ring. The power of the radius when the ring is formed (r_b^2) is assigned to the x-coordinate as the birth value. At a larger radius ($r = r_d$), this ring structure is filled by the spheres. Here, r_d^2 is used as death value and assigned to the y-coordinate.

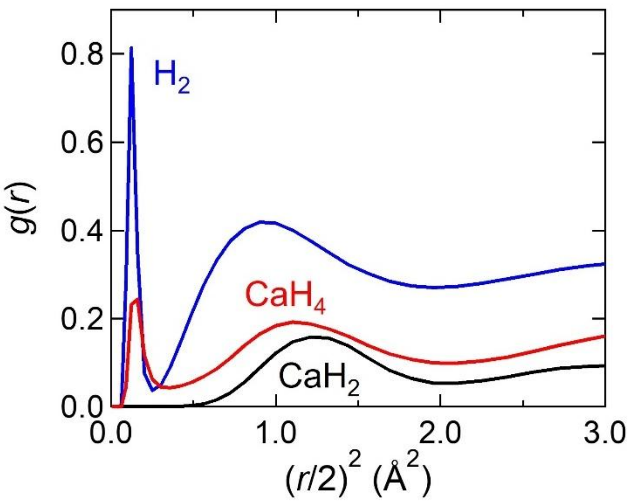

Fig. S21. Pair distribution function (PDF) of H atoms around the H atom during 100-ps MLP-MD simulations for CaH_2 (black) and CaH_4 (red), and H_2 (blue) bulks at 1500K under 40 GPa. Here, $(r/2)^2$ has been chosen for the horizontal axis so that it can be easily compared with the birth values in persistence diagram in Fig. 2 and Fig. S3.

A
B

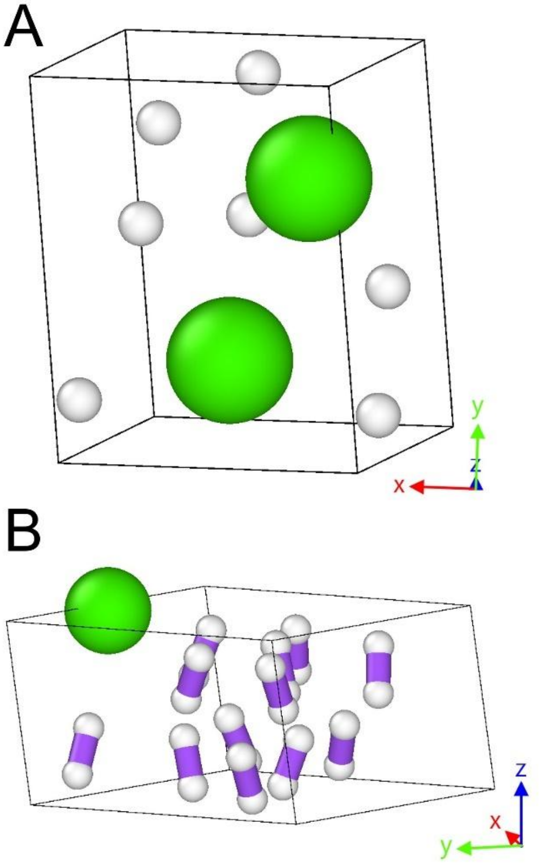

Fig. S22. Atomic configurations of (A) $CaH_{3.5}$ and CaH_{24} predicted from USPEX calculations.

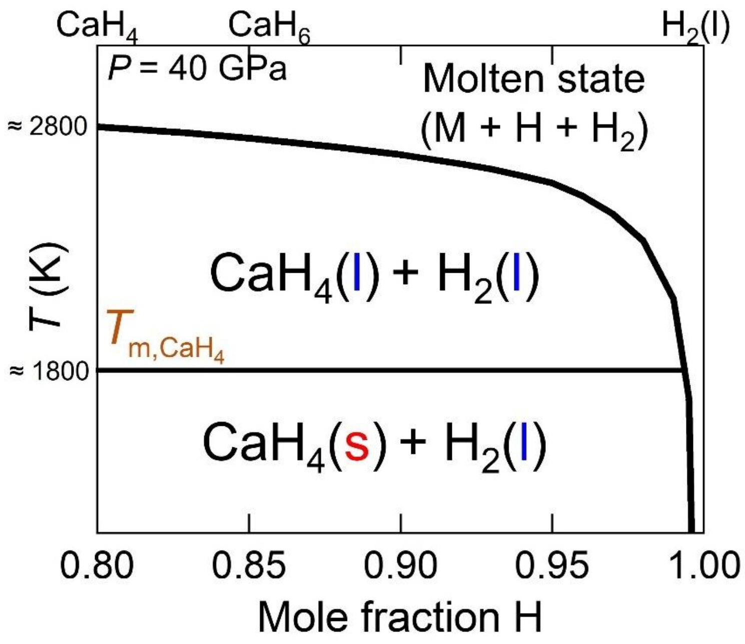

Fig. S23. Ca-H phase diagram at 40 GPa constructed from the stable structures and MLP-MD simulation results.

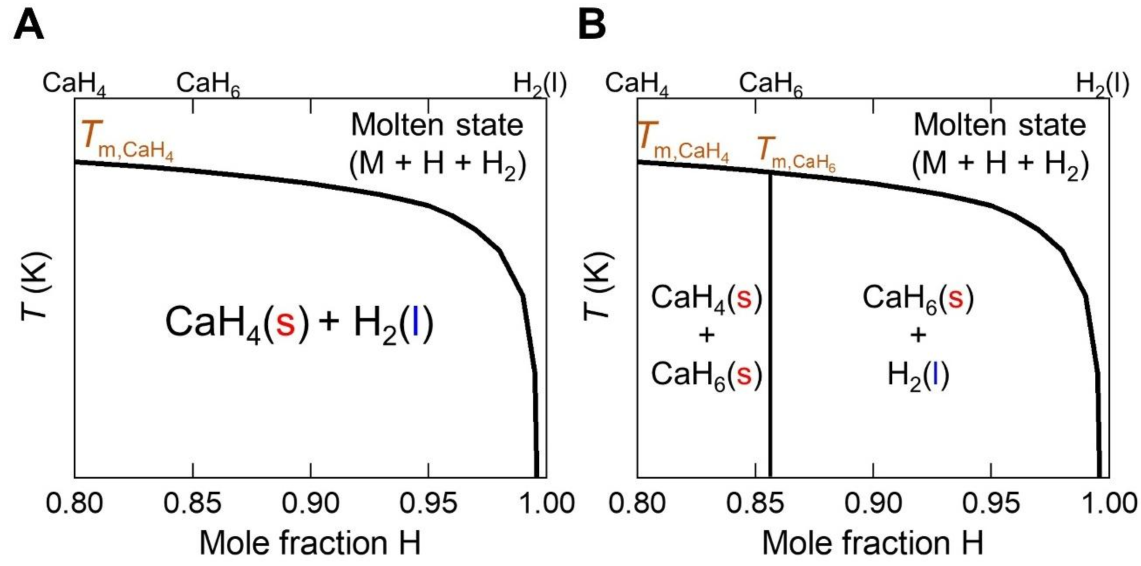

Fig. S24. The Hypothetical phase diagrams of Ca-H presuming (A) no liquid CaH_4 and (B) the existence of CaH_6 without considering both liquid CaH_4 and CaH_6.

Table S1. List of CaH_4 synthesis conditions of the MLP-MD simulation (this work) and previous studies (***17-19***). Note that ref. ***17–19*** in this supplementary information is identical to ref. ***33–35*** in the main article.

	P (GPa)	*T* (K)
This work (MLP-MD)	60 (rescaled with DFT enthalpy)	1500
A. K. Mishra et. al. JPCC (2018) (***17***)	116	1600
G. Wu el. al. JCP (2019) (***18***)	48.5	1900
M. Peña-Alvarez JPCL (2022) (***19***)	50	1600

Table S2. List of DFT and AIMD simulations used to construct MLP and validate the accuracy of MLP.

**System (number of atoms)**	***T* (K)**	***P* (GPa)**	**Time (Δt=0.5 fs)**	**MAE (MLP vs DFT) (eV/Å)**
Ca (Ca 64)	1000	0	0.6 ps	0.02
Ca (Ca 64)	3000	0	0.6 ps	0.05
CaH_2(Ca 16 H 32)	1000	0	2.5 ps	0.06
CaH_2(Ca 16 H 32)	3000	0	2.5 ps	0.12
CaH_2(Ca 16 H 32)	10000	0	1.3 ps	—
CaH_4(Ca 16 H 64)	1000	25	2.5 ps	0.13
CaH_4(Ca 16 H 64)	3000	25	2.5 ps	0.25
CaH_4(Ca 16 H 64)	10000	25	2.5 ps	—
CaH_6 (Ca 16 H96)	1000	150	2.5 ps	0.26
CaH_6 (Ca 16 H96)	3000	150	2.5 ps	0.35
CaH_6 (Ca 16 H96)	10000	150	2.5 ps	—
H_2 (H 32) ※*k* point = 8*8*4	1000	0	0.8 ps	—
**Interface with small number of H_2 molecules**				
Ca+H_2	1000	150	1.85 ps	0.60
CaH_2+H_2 (Ca 16 H 64)	1000	50	1.5 ps	0.20
CaH_4+H_2 (Ca 16 H 96)	1000	150	2.1 ps	0.34
**Geometrical optimization starting from 80% and 120% lattice parameters**				
Ca (Ca 64)	—	0	—	
CaH_2(Ca 16 H 32)	—	0	—	
CaH_4(Ca 16 H 64)	—	0	—	
CaH_6 (Ca 16 H96)	—	0	—	

**Structure after geometrical optimization with various *P* (GPa)**				
Ca (Ca 64)	—	0, 10, 20, 30, 40, 50, 60, 70, 80, 90, 100, 125, 150 (Ca only ~ 50)	—	
CaH_2(Ca 16 H 32)	—		—	
CaH_4(Ca 16 H 64)	—		—	
CaH_6 (Ca 16 H96)	—		—	
H_2 (H 96)	—		—	
**AIMD (not included in training data)**				
CaH_2+H_2 (Ca 32 H 320)	1000	30	1.5 ps	0.34
CaH_4+H_2 (Ca 32 H 384)	1000	50	1 ps	0.42
H_2 (H 256)	1000	10	2.5 ps	0.13
H_2 (H 256)	1000	25	2.5 ps	0.13
H_2 (H 256)	1000	50	2.5 ps	0.15
H_2 (H 256)	1000	75	2.5 ps	0.19
H_2 (H 256)	1000	100	2.5 ps	0.25
H_2 (H 256)	1000	125	2.5 ps	0.34
H_2 (H 256)	1000	150	2.5 ps	0.63
**Single-step calculation using MLP-MD configurations (not included in training data)**				
CaH_2(100)+H_2 (Ca 64 H 640)	300-1500 (20 ps) 1500 (30 ps) + 1500-300 (30 ps)	10 to 50 (20 ps) + 50 (60 ps)	800 configurations (80 ps)	0.21
CaH_4(101)+H_2 (Ca 64 H 640)	300-1500 (20 ps) 1500 (100 ps)	30 to 10 (20 ps) + 10 (100 ps)	1200 configurations (120 ps)	0.19

Table S3. List of CaH_2 surface energy under vacuum condition used for Wulff shape calculation.

Miller index	Surface energy (eV/Å^2)
(100)	0.041
(010)	0.034
(001)	0.068
(012)	0.041
(103)	0.043

Table S4. Atomic configuration of $CaH_{3.5}$ predicted from USPEX calculation

CaH3.5 Sym.group=8		
1		
3.29805	0.00366	-0.05126
0.473906	4.323852	-0.05657
-1.05005	0.139385	2.464744
Ca	H	
2	7	
Direct		
0.133319	0.742266	0.134129
0.505791	0.240452	0.31714
0.047197	0.410259	0.588801
0.124929	0.070612	0.630389
0.504635	0.923627	0.818454
0.922321	0.162344	0.023104
0.824312	0.566158	0.481837
0.585153	0.549805	0.859335
0.721425	0.835248	0.428667

Table S5. Atomic configuration of CaH_{24} predicted from USPEX calculation

CaH24_Sym.group=5		
1		
2.2564	-3.82588	-0.11679
2.191861	3.867525	0.039761
-1.15292	0.680016	3.317887
Ca	H	
1	24	
Direct		
0.50277	0.997019	0.962224
0.098651	0.066946	0.598034
0.032855	0.179506	0.111949
0.111444	0.893559	0.158001
0.734871	0.189546	0.552929
0.769516	0.796906	0.343129
0.774442	0.824578	0.821782
0.046764	0.210985	0.332849
0.467152	0.33629	0.593864
0.959381	0.664243	0.583164
0.644863	0.813161	0.597408
0.414489	0.351548	0.097473
0.891121	0.495596	0.097022
0.971065	0.653974	0.804293
0.536902	0.362745	0.31789
0.360047	0.660839	0.32749
0.230904	0.533679	0.582047
0.231974	0.553752	0.101924
0.272134	0.495471	0.801838
0.584924	0.439224	0.814578
0.903728	0.464609	0.316729
0.111059	0.104613	0.816772
0.736105	0.726403	0.126283
0.273456	0.952234	0.372359
0.893637	0.286942	0.770235

Movie S1 (separate file). Movie of atomic configurations during MLP-MD simulation for CaH_2(100)/H_2 interface at 1500K under 40 GPa.

SI References

1. K. Shimamura *et al.*, Guidelines for creating artificial neural network empirical interatomic potential from first-principles molecular dynamics data under specific conditions and its application to α-Ag2Se. *J. Chem. Phys.*, **151**, 124303 (2019).
2. B. Cheng, G. Mazzola, C. J. Pickard, M. Ceriotti, Evidence for supercritical behaviour of high-pressure liquid hydrogen. *Nature*, **585**, 217–220 (2020).
3. B. W. Hamilton *et al.*, High-pressure and temperature neural network reactive force field for energetic materials. *J. Chem. Phys.*, **158**, 144117 (2023).
4. D. Unruh *et al.*, Gaussian approximation potential for amorphous Si : H. *Phys. Rev. Mater.*, **6**, 065603 (2022).
5. X.-Y. Wang *et al.*, Deep neural network potential for simulating hydrogen blistering in tungsten. *Phys. Rev. Mater.*, **7**, 093601 (2023).
6. Y. Hiraoka *et al.*, Hierarchical structures of amorphous solids characterized by persistent homology. *Proc. Natl. Acad. Sci. U. S. A.*, **113**, 7035–7040 (2016).
7. A. Hirata, T. Wada, I. Obayashi, Y. Hiraoka, Structural changes during glass formation extracted by computational homology with machine learning. *Commun. Mater.*, **1**, 98 (2020).
8. R. Sato *et al.*, Topological data analysis of ion migration mechanism. *J. Chem. Phys.*, **158**, 144116 (2023).
9. I. Obayashi, T. Nakamura, Y. Hiraoka, Persistent homology analysis for materials research and persistent homology software: HomCloud. *J. Phys. Soc. Jpn.*, **91**, 091013 (2022).
10. I. Obayashi, Stable volumes for persistent homology. *J Appl. Comput. Topol.*, **7**, 671–706 (2023).
11. A. R. Oganov, C. W. Glass, Crystal structure prediction using *ab initio* evolutionary techniques: Principles and applications. *J. Chem. Phys.*, ***124,*** 244704 (2006).
12. A. R. Oganov, A. O. Lyakhov, M. Valle, How Evolutionary Crystal Structure Prediction Works—and Why. Acc. Chem. Res., ***44,*** 227-237 (2011).
13. A. O. Lyakhov, A. R. Oganov, H. T. Stokes, Q. Zhu, New developments in evolutionary structure prediction algorithm USPEX. Comput. Phys. Commun., ***184***, 1172-1182 (2013).
14. P. V. Bushlanov, V. A. Blatov, A. R. Oganov, Topology-based crystal structure generator. Comput. Phys. Commun., ***236***, 1-7 (2019).
15. H. Wang *et al.*, Superconductive sodalite-like clathrate calcium hydride at high pressures. *Proc. Natl. Acad. Sci. U. S. A.*, **109**, 6463−6466 (2012).
16. C. M. Tenney, Z. F. Croft, J. M. McMahon, Metallic Hydrogen: A Liquid Superconductor?, *J. Phys. Chem. C*, ***125***, 23349-23355 (2021).
17. A. K. Mishra *et al.*, New Calcium Hydrides with Mixed Atomic and Molecular Hydrogen, *J. Phys. Chem. C*, ***122***, 19370-19378 (2018).
18. G. Wu *et al.*, Unexpected calcium polyhydride CaH_4: A possible route to dissociation of hydrogen molecules, *J. Chem. Phys.*, ***150***, 044507 (2019).
19. M. Peña-Alvarez *et al.*, Chemically Assisted Precompression of Hydrogen Molecules in Alkaline-Earth Tetrahydrides, *J. Phys. Chem. Lett.*, ***13***, 8447‑8454, (2022).
